# Integrated Study of Fluorescence Enhancement in the Y176H Variant of Cyanobacterial Phytochrome Cph1

**DOI:** 10.1021/acs.biochem.4c00687

**Published:** 2025-02-27

**Authors:** Soshichiro Nagano, Chen Song, Valentin Rohr, Megan J. Mackintosh, Oanh Tu Hoang, Anastasia Kraskov, Yang Yang, Jon Hughes, Karsten Heyne, Maria-Andrea Mroginski, Igor Schapiro, Peter Hildebrandt

**Affiliations:** 1 Institute for Plant Physiology, Justus Liebig University, Senckenbergstr. 3, Giessen D-35390, Germany; 2 Institute for Analytical Chemistry, University of Leipzig, Johannisallee 29, Leipzig D-04103, Germany; 3 Fritz Haber Center for Molecular Dynamics, Institute of Chemistry, Hebrew University of Jerusalem, Jerusalem 91904, Israel; 4 Institute for Chemistry, Technical University of Berlin, Str. des 17. Juni 135, Berlin D-10623, Germany; 5 Department of Physics, Free University of Berlin, Arnimallee 14, Berlin D-14195, Germany

## Abstract

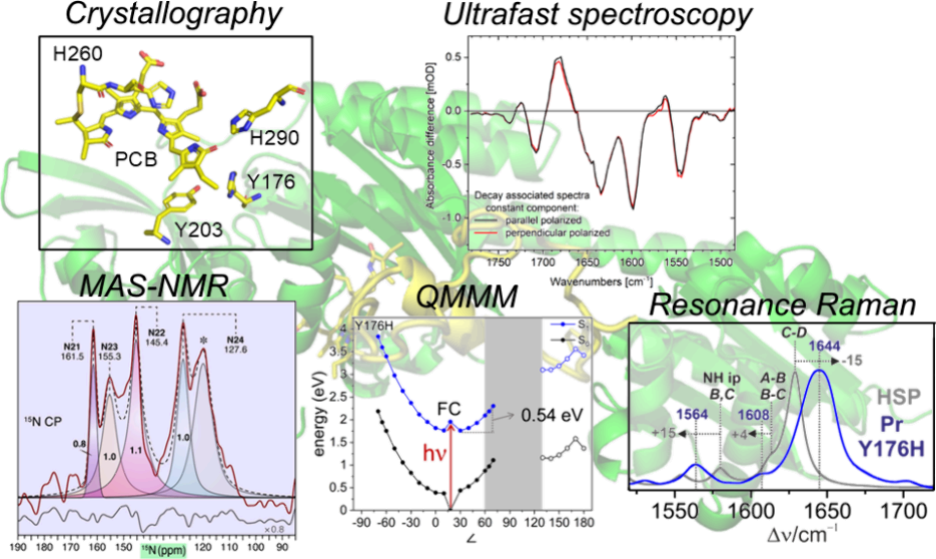

Phytochromes are red-light-sensitive biliprotein photoreceptors that control a variety of physiological processes in plants, fungi, and bacteria. Lately, greater attention has been paid to these photoreceptors due to their potential as fluorescent probes for deep-tissue microscopy. Such fluorescing phytochromes have been generated by multiple amino acid substitutions in weakly fluorescent wild-type (WT) proteins. Remarkably, the single substitution of conserved Tyr176 by His in cyanobacterial phytochrome Cph1 increases the fluorescence quantum yield from 2.4 to 14.5%. In this work, we studied this Y176H variant by crystallography, MAS NMR, resonance Raman spectroscopy, and ultrafast absorption spectroscopy complemented by theoretical methods. Two factors were identified to account for the strong fluorescence increase. First, the equilibrium between the photoactive and fluorescent substates of WT Cph1 was shown to shift entirely to the fluorescent substate in Y176H. Second, structural flexibility of the chromophore is drastically reduced and the photoisomerization barrier is raised, thereby increasing the excited-state lifetime. The most striking finding, however, is that Y176H includes the structural properties of both the dark-adapted Pr and the light-activated Pfr state. While the chromophore adopts the Pr-typical *ZZZssa* configuration, the tongue segment of the protein adopts a Pfr-typical α-helical structure. This implies that Tyr176 plays a key role in coupling chromophore photoisomerization to the sheet-to-helix transition of the tongue and the final Pfr structure. This conclusion extends to plant phytochromes, where the homologous substitution causes light-independent signaling activity akin to that of Pfr.

## Introduction

Phytochromes are red/far-red photochromic proteins containing linear methine-bridged tetrapyrrole chromophores. The photoreceptors are found in plants and widespread among microorganisms, controlling numerous physiological processes.^1^ In prototypical phytochromes, photoexcitation of the dark-adapted Pr parent state leads to chromophore isomerization at the methine bridge between rings C and D, followed by thermal relaxations and conformational changes in the protein, eventually generating the light-activated Pfr state. The underlying structural changes include movement of numerous side chains and sheet-to-helix restructuring of a tongue-like hairpin extension of the PHY-domain.^2,3^ The photochemical quantum yield (Φ_ph_) of the Pr → Pfr phototransformation is typically around 15%,^4^ such that the remaining excited molecules of Pr fall back to the ground state by dissipating their energy either thermally or via fluorescence. Although the fluorescence quantum yield (Φ_f_) of Pr rarely exceeds 2% in WT phytochromes at ambient temperature, this fluorescence is of interest in the context of *in vivo* labeling on account of their extraordinarily high extinction coefficients (∼100 mM^–1^ cm^–1^) at long wavelengths (>650 nm) where scattering is reduced.^5^ In bacteriophytochromes carrying a biliverdin cofactor, multiple amino acid substitutions have increased Φ_f_ 10-fold or more, paving the way toward applications in deep-tissue microscopy.^6−11^ Interestingly, however, the cyanobacterial phytochrome Cph1 from *Synechocystis* 6803^12,13^ shows an increase of Φ_f_ by a factor of ca. 6 following a single Tyr → His (Y176H) substitution.^14^ Hence, Y176H may be a starting point for generating further fluorescence-optimized variants preferentially via rational design principles.

The WT Pr state was shown to be structurally heterogeneous, with two substates (Pr–I and Pr–II) forming a pH-dependent equilibrium with a p*K*_a_ of 7.5.^15−18^ The substates differ regarding the protonation state of the conserved His260, which is double-protonated (cationic) in Pr–I (< pH 7.5), but single-protonated in Pr-II.^16,17^ A second conserved His (His290) remains single-protonated in both substates. These protonation patterns are detectable by solid-state magic-angle spinning (MAS) nuclear magnetic resonance (NMR) and, due to their effect on the chromophore structure, also by resonance Raman (RR) spectroscopy. Pr–I corresponds to the species that was crystallized (PDB code2VEA)^19^ as shown by the identity of the RR spectra of the corresponding Cph1 crystals and pure Pr–I in solution.^16,20^ The electronic properties of the chromophore are different in the two substates.^16,18^ The increased proportion of Pr–II at a higher pH is reflected by a blue-shift of the lowest energy electronic transition. Since the maximum of the fluorescence excitation spectrum at 643 nm is distinctly lower than the absorption maximum at pH 7.8 (658 nm)^21^ where both substates coexist in nearly equal portions, we conclude that Φ_f_ of Pr–II is considerably greater than the 2.4% determined for the WT Pr–I/Pr-II mixture.^21−23^ Remarkably, the overall fluorescence increase in Y176H^14,21,24^ is also observed in plant phytochrome^14^ for the homologous substitution but not in bacteriophytochromes.^25^ Interestingly, in plant phytochromes, this single mutation has dramatic physiological consequences: Transformation of the *Arabidopsis phyB-5* null mutant with *AtPHYB*^*Y276H*^ results in transgenic seedlings that exhibit constitutively photomorphogenic (cop) development in total darkness.^26^ The substitution thus causes structural changes that affect both the fluorescence yield of the chromophore and the functionally important conformational properties of the protein.

In this work, we have addressed both issues on the basis of the crystal structure of the Y176H variant of the Cph1 photosensory module as Pr. Details of the chromophore structure and the protonation pattern of the surrounding amino acids were revealed by MAS NMR and RR spectroscopy, which also supported the generation of a structural model from the X-ray diffraction data. The crystal structure and additional NMR data provided the basis for a geometry-optimized structural model of the chromophore binding pocket (CBP) in the ground state (S_0_) by molecular dynamics (MD) simulation and quantum mechanics/molecular mechanics (QMMM) techniques. This model was then validated by RR spectroscopy. The calculations were subsequently extended to the excited state (S_1_), which together with ultrafast transient absorption spectroscopy contributed to an understanding of the energy dissipation pathways.

## Methods

### Mutagenesis, Protein Production, and Purification

The mutations coding for Y176H and Y272H (Uniplot Q55168 and I1MGE5, respectively) were introduced into the expression constructs of the WT Cph1 photosensor (residues 1–514, comprising the NTS, nPAS, GAF, and PHY domains)^19^ and *Gm.*phyB NPGP (N-terminal extension, nPAS, GAF and PHY domains),^27^ respectively, using the back-to-back primer method (Table S1). Specifically for crystallization of Y176H, the N141R mutation was introduced to promote dimerization,^28^ and also the 6-His-tag was relocated to the N-terminus followed immediately by a TEV cleavage sequence. Recombinant holophytochromes carrying phycocyanobilin (PCB) were produced *in vivo*,^29^ unless otherwise specified and purified as described.^19,27^ For crystallization, Cph1 Y176H was additionally treated overnight with TEV protease to cleave the N-terminal 6-His-tag that was then removed by Ni-affinity prior to size exclusion chromatography using an ÄKTA FPLC system and a 16/60 Superdex 200 column (GE Healthcare). Y176H holoprotein for MAS NMR was produced by *in vitro* assembly with [^13^C,^15^N]-PCB as described,^17^ using the p926.5 plasmid bearing the Y176H mutation.

## Crystal Structure Determination

Crystallization screening using an Oryx4 robot (Douglas Instruments) yielded needle-like crystals of Cph1 Y176H in 0.3 M ammonium formate, 0.1 M HEPES (4-(2-hydroxyethyl)-1-piperazineethanesulfonic acid) pH 7.0, 20% (w/v) Sokalan CP 5. These crystals were crushed and used as seeds to generate crystals more suitable for X-ray diffraction analysis in 0.3 M ammonium formate, 0.1 M HEPES pH 6.9, and 18% Sokalan CP7. The crystals were dehydrated in 75% (v/v) crystallization solution and 25% (v/v) of 70% (w/v) xylitol in open air for 18 h and then vitrified in liquid nitrogen. The crystal structure was determined as before.^27^ The crystallographic phase was solved by molecular replacement with PHASER^30^ using the 2VEA structure split into nPAS-GAF and PHY (without the tongue, residues 443–487) fragments (Table S2). The most probable side-chain rotamer of the functionally important His176 residue was derived by MD simulations, which allowed two alternative ring orientations to equilibrate. Regardless of the initial ring orientations, the side-chain rotamer converged to the m-70° rotamer^31^ in 80 ns, which was thus used in the deposited model. The molecular structure was visualized using PyMOL and superposed using the Super command with the default settings.

### MAS NMR Spectroscopy

All NMR experiments were carried out on a narrow-bore Bruker AVANCE NEO 600 spectrometer (Rheinstetten, Germany) equipped with a 3.2 mm double-resonance MAS probe at −30 °C under a MAS rate of 15 kHz. 2D ^13^C–^13^C dipolar-assisted rotational resonance (DARR) and ^1^H–^13^C/^15^N heteronuclear correlation (HETCOR) experiments were used to determine the ^13^C, ^15^N, and ^1^H chemical shifts of the chromophore atoms and to identify the protein–chromophore interactions.^32^ Detailed acquisition and processing parameters are described in the Supporting Information (section 1.1).

### Resonance Raman Spectroscopy

RR measurements were performed using a Bruker Fourier-transform Raman spectrometer RFS 100/S with 1064 nm excitation (Nd:YAG cw laser, line width 1 cm^–1^), equipped with a nitrogen-cooled cryostat from Resultec (Linkam). All spectra of the samples in frozen solution were recorded at ca. 90 K with laser power at the sample of 690 mW and a typical accumulation time of 1 h. In order to identify potential laser-induced damage to the samples, RR spectra before and after a series of measurements were compared. Protein and buffer Raman bands were subtracted on the basis of the Raman spectrum of the apoprotein. Background subtraction was carried out by using the OPUS software (Bruker).

### Ultrafast Spectroscopy

The Y176H variant samples in D_2_O (pD 7.8) had an optical density of ∼0.5 at 660 nm. The samples were excited at 635 nm and probed in the mid-IR spectral range. The detail of the setup is reported elsewhere.^33^ We used polarization-resolved femtosecond visible (VIS) pump infrared (IR) probe spectroscopy to investigate the dynamics of the photoreaction under isotropic and polarization-resolved conditions. The sample was moved continuously in horizontal and vertical directions by a Lissajous-scanner to ensure sample exchange between two successive pump pulses. All experiments were performed at room temperature. Further details are given in the Supporting Information (section 1.2).

### MD Simulations and Quantum Mechanical Calculations

The computational model was generated from chain A of the Y176H crystal structure of Cph1 reported in this work. We also created a model of the Cph1 WT (PDB ID: 2VEA). The crystal waters from the WT were retained for all simulations, while in the structure of Y176H, no waters were modeled, except for the pyrrole water. The first 20 missing residues in Y176H were added as an α-helix using the USCF Chimera program. Hydrogen were added using the *tleap* program in AMBER16 where the protonation state of the titratable residues was considered at pH 7.0. The conserved His residues near the PCB chromophore were protonated as follows. For His260, Nε was protonated (HIE260). For His290, both Nε (HIE290) and Nδ (HID290) were tested. For Y176H, protonation at the Nε (HIE176) and Nδ (HID176) positions was tested. These three models were treated by QMMM and MD simulations as described in detail in the Supporting Information (section 1.3).

## Results

### Overall Structure of the Protein

**Figure 1:**
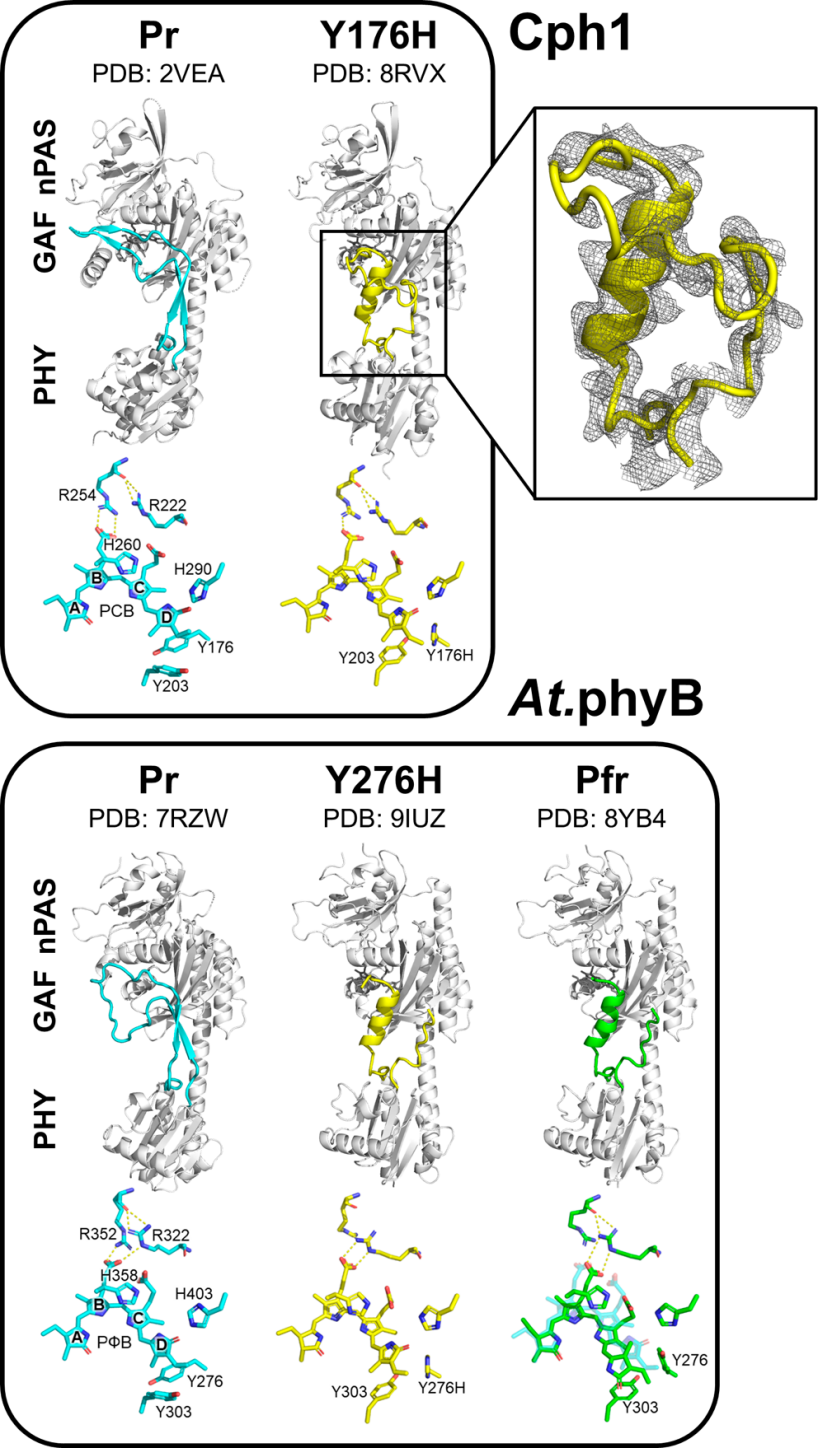
3D structures of Cph1 (WT Pr—PDB code 2VEA; Y176H—PDB code 8RVX) and *At*.phyB (WT Pr—PDB code 7RZW; Y276H—PDB code 9IUZ; Pfr—PDB code 8YB4). Upper rows: Structures of the N-terminal photosensory module with the PHY-domain tongue are highlighted by different colors. The PHY-domain tongue features antiparallel β-sheet(s) and α-helix in Pr and Pfr, respectively. The Tyr → His variants of Cph1 and *At*.phyB both show a Pfr-like α-helical tongue structure. The electron density map (2Fo–Fc contoured at 1.0 rmsd) is shown for the PHY-tongue of Cph1 Y176H. Lower rows: Chromophore and adjacent residues. The bilin adopts the 15Z and 15E configuration in Pr and Pfr, respectively. Correspondingly, the “tyrosine dyad” exhibits different conformations in Pr and Pfr. The variants, however, show the Pr-like 15Z bilin configuration and Pfr-like conformation at the tyrosine dyad. The chromophore in Pr (translucent cyan) is overlaid onto that of *At*.phyB in Pfr to illustrate the flip-and-rotate motion.

The crystal structure of Y176H was determined at 3.7 Å resolution (PDB code 8RVX; Figure 1 and Table S2), the four protomers in the asymmetric unit comprising two antiparallel dimers (chains A-B and C-D). Protomer A was modeled contiguously, whereas residues 453–465 (chain B), 73–80 (chain C), and 452–465 (chain D) could not be modeled due to sparse electron densities, probably reflecting local disorder.

These disordered segments correspond to regions that have no clearly defined secondary structure, as judged from Y176H chain A or related regions of WT Cph1 (73–80) and the F469W variant of *Dr*BphP (∼453–463). Despite overall similar structures, the root-mean-square deviations (rmsds) between the chains are 1.0–1.9 Å following structural superposition of all four protomers. The variability results from localized structural deviations, e.g., between α2 and α3 of the nPAS domain^19^ and variations in interdomain orientations.

Y176H shares characteristic structural properties with other phytochromes. For example, the knot around the nPAS domain involving the GAF domain and the N-terminal extension, the solvent shield around the chromophore, and its covalent attachment to GAF domain Cys259 are all conserved among cyanobacterial and plant phytochromes. The position of the chromophore with respect to the binding pocket is very similar in Y176H and the WT (PDB: 2VEA) (Figure 1). This is in contrast to those cases where the relative orientation between the chromophore and the CBP differ between Pr and Pfr due to the “flip-and-rotate” motion associated with the photoconversion.^34−38^ Solely based on the electron densities, the structure of the chromophore cannot be determined unambiguously at the present crystallographic resolution. Thus, the overall PCB geometry was modeled as *ZZZssa* following the NMR and RR spectroscopic data as discussed in the following sections. NMR spectroscopy also suggested the D-ring ethyl group orientation toward His176. The D-ring itself was assumed to be tilted toward Tyr263, corresponding to an α-facial disposition. This disposition, which avoids steric clashes with nearby amino acids, is in line with the circular dichroism spectrum of Y176H that is similar to that of the Pr state of WT Cph1^21^ and other phytochromes.^1^

**Figure 2:**
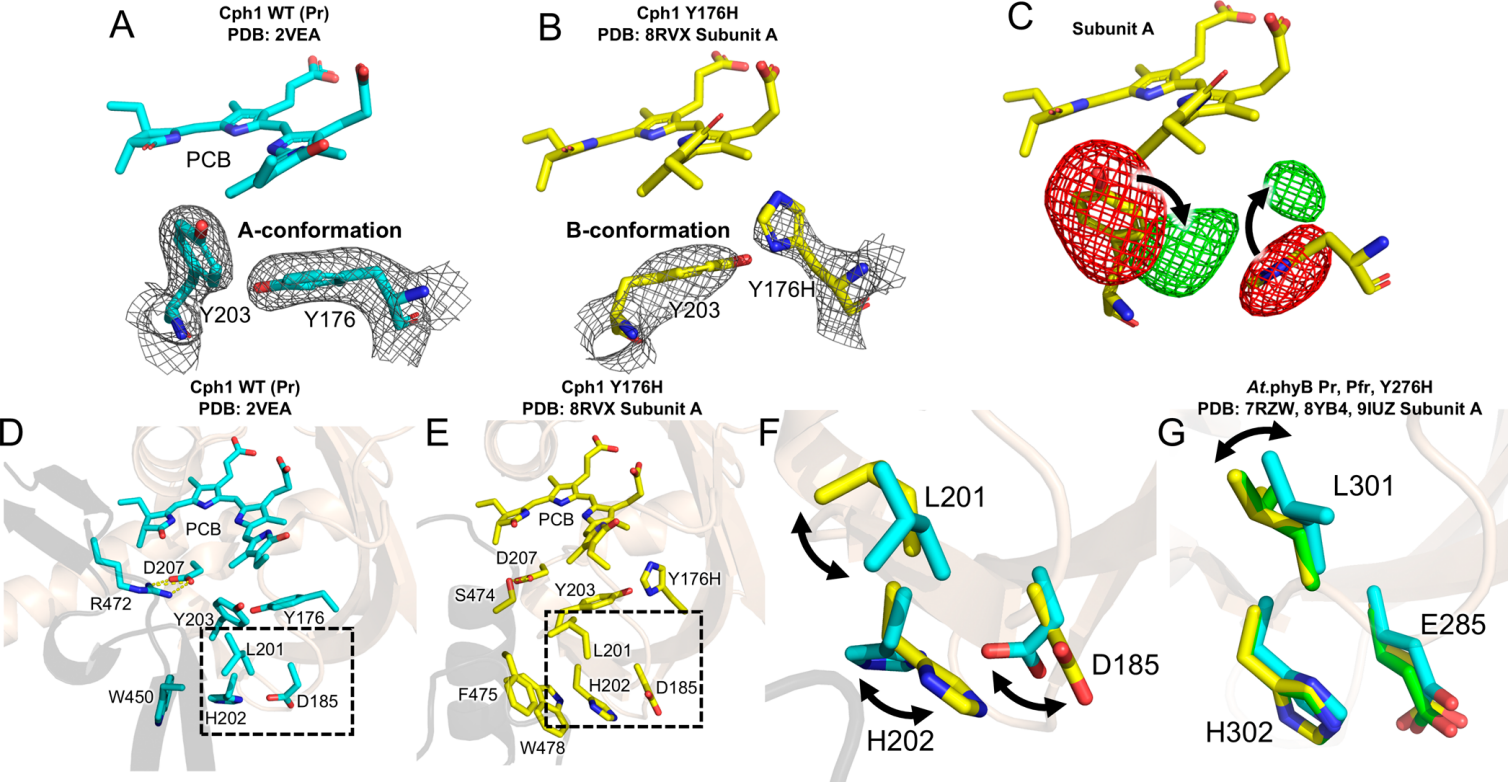
Structural details of Cph1 Y176H and *At*.phyB Y276H in comparison with WT Pr and Pfr. The Pfr-like *B*-conformation in Y176H panel (B) is evidenced by the electron density map (2Fo – Fc, 1.5 rmsd contour) around the “tyrosine dyad”. This dyad contracts in the *A*-conformation found in the WT in Pr (A). No difference signal (Fo-Fc) is identified at the 3.5 rmsd contour level. (C) Refinement of the Y176H structure with the tyrosine dyad in the WT-like *A*-conformation results in difference signals (Fo–Fc, 3.5 rmsd contour level) and support the *B*-conformation (indicated by black arrows). Similar difference signals are noted in all of the other subunits (Figure S1). (D–F) The chromophore and key amino acids are shown (stick representation), with the remainder of the protein presented as cartoon (GAF: beige, PHY-tongue: black). The conformational difference at the tyrosine dyad (Tyr/His176 and Tyr203) is accompanied by conformational differences between Pr and Y176H at Asp185, Leu201, and His202 (in the dotted boxes in panels (D) and (E) and enlarged view in panel (F); cyan, Pr; yellow, Y176H). Similar differences are observed in all of the other subunits (Figure S1). This structural difference is associated with the tryptophan switch, where the space occupied by Trp450 of the WGG motif in Pr is instead occupied by Trp478 and Phe475 of the PRxSFxxW motif in Y176H. These structural differences ultimately result in a drastic difference at the PHY-tongue (Figure 1). (G) Conformations of equivalent residues in *At*.phyB structures (cyan, Pr; green, Pfr; yellow, Y276H). Superposition of subunit B reveals similar conformational patterns (not shown).

Based on the spectroscopy-assisted structural model, we can now inspect the CBP and its environment in more detail. Most surprisingly, we observe the characteristic properties of both Pr and Pfr, which we denote as the *A*- and *B*-conformations, respectively. Whereas the interactions of conserved Arg222 and Arg254 with the B-ring propionate are similar to those of the WT Pr (*A*-conformation), a drastic change is observed at the Tyr → His mutated 176 site and its neighbor Tyr203, corresponding to the Pfr-characteristic *B*-conformation (Figure 2).

Even though the resolution of 3.7 Å is modest, refinement with the Pr-like *A*-conformation in this region resulted in substantial difference signals (Figure S1). The B-conformation involves concerted changes at Asp185, Leu201, His202, and finally, a “tryptophan swap”,^2^ where the space occupied by Trp450 in WT Pr is replaced by Trp478 and Phe475 in Y176H (Figure 2). Less drastic conformational differences are found in Pr, Pfr, and Y276H of *At*.phyB, however, despite high sequence conservation, pointing toward nonidentical signal transduction mechanisms despite similar outcomes of an α-helical PHY-tongue in Pfr and the YH mutant (Figure 2G). Pfr-like structural features of Y176H also include the interaction between conserved Asp207 of the PASDIP motif and Ser474 of the PRxSF motif. This contrasts the Pr-characteristic salt bridge between Asp207 and Arg472 (Figure 2). These interactions stabilize the α-helical structure of the PHY-tongue (residues 471–482; Figure 1 and Figure S1)—the hallmark of Pfr clearly observed in all four chains.

### Structural Details of the CBP

**Figure 3:**
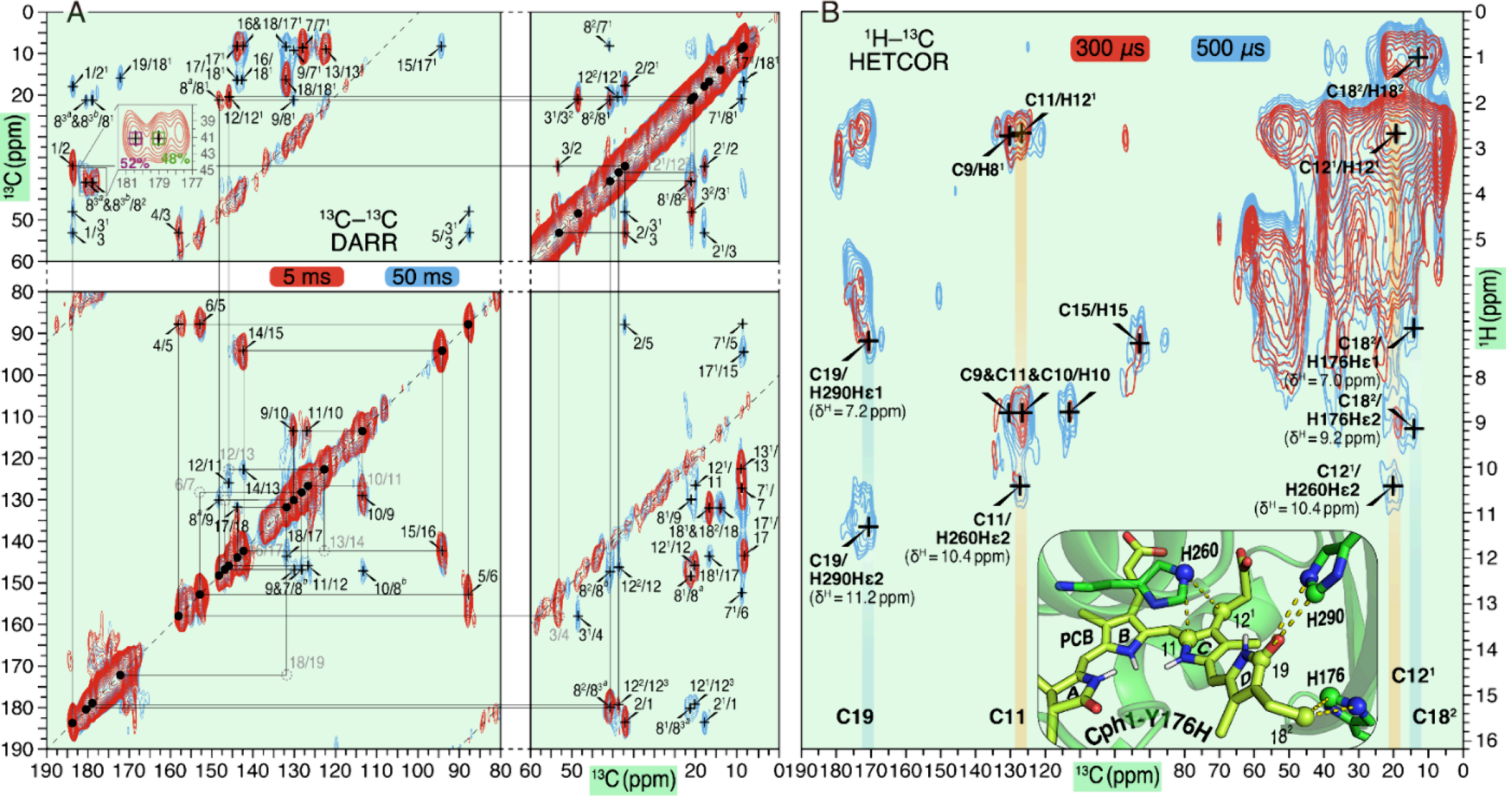
MAS NMR experiments on the Y176H mutant of the Cph1 sensory module, assembled with doubly ^13^C- and ^15^N-labeled PCB (*u*-[^13^C,^15^N]-PCB-Cph1-Y176H) as Pr. (A) Overlay of 2D ^13^C–^13^C DARR spectra acquired with proton mixing times of 5 (red) and 50 ms (cyan). All carbon pairs of the PCB chromophore are indicated by arrows and labeled selectively (see Table S3 and Figures S2 and S3 for full spectra with complete assignments). Observed ^13^C signal doubling of C8^3^ are superscripted with *a* and *b* from the low- to high-field side. (B) Overlay of 2D ^1^H–^13^C HETCOR spectra acquired with CP contact times of 300 (red) and 800 μs (cyan). The spectra reveal both intramolecular CH correlations of the PCB chromophore and interfacial correlations between PCB carbons and protons from unlabeled protein residues in close proximity. The tentative assignment of ^1^H interfacial correlations was based on the corresponding crystal structure of this mutant. The structural view showing the chromophore and its surrounding residues with observable ^1^H contacts is *inset*. The yellow dotted lines indicate the observed ^1^H_resi._–^13^C_PCB_ correlations.

Structural details and information about the protonation pattern of the chromophore and its immediate environment were obtained by MAS NMR and RR spectroscopy. Using a similar strategy as for the WT Pr,^17^ we derived complete ^13^C and ^15^N assignments for the NMR spectra of the doubly labeled PCB chromophore within the Y176H holoprotein from a series of 2D correlation experiments. More specifically, the assignment of PCB carbons is obtained from the well-defined correlation network (Figure S2, indicated by gray lines). The correlation peak from the ethyl side chain of ring D (C18^1^–C18^2^, dashed line) is clearly present by using a short proton mixing time of 5 ms, and the assignment of C18^2^ is confirmed by a two-bond C18–C18^2^ correlation resolved in the spectrum recorded with a mixing time of 50 ms (see Figure S2 for complete assignments). Strikingly, almost all PCB carbon atoms showed a single correlation network (Figure 3A and Figure S2), with the exception of the B-ring propionate carboxylate carbon (C8^3^), for which the resonance is weakly split into two with a separation of ∼1.5 ppm, most likely due to local dynamics (Figure S2). By contrast, WT Pr showed widespread resonance splitting as reported earlier.^17^ These results suggest that the overall rigidity of the Pr chromophore in the binding pocket is greatly enhanced by the Y176H mutation.

Prominent δ^C^ changes associated with Y176H substitution are seen for the methine bridge anchors between rings A-B (C4, C6) and C-D (C14, C16) (Figure S4) and point to a reduced conjugated bilin system, probably related to modified A- and D-ring geometries. The ^1^H correlation of D-ring N24 is less resolved due to the partial overlap with the amide resonances originating from the protein backbone in natural abundance and is thus not included in Figure S3. The low intensity of this correlation could arise through changes in the local hydrogen-bonding/hydrophobic environment or due to molecular motion of the D-ring NH group. ^1^H–^13^C correlation spectra (Figure 3B) provided one- and two-bond intramolecular correlations of the chromophore itself (e.g., C12^1^/C11–H12^1^) and interfacial contacts between the chromophore carbons and the protons bound to nearby residues.

The close proximity of His176 to the D-ring ethyl side chain (Figure 3B, inset) agrees with correlations of the imidazole protons (Hε1 and Hε2 with δ^H^ of 7.0 and 9.2 ppm, respectively) and C18^2^ and thus served as an input for modeling the crystallographic structure. The His260 residue α-facial of the B- and C-rings shows correlations of ring C atoms C11 and C12^1^ with the imidazole Hε2 proton (δ^H^ = 10.4 ppm) at a distance of ∼3.9 Å (C11/C12^1^···Nε2), nearly identical to that of the Pr–II substate of the WT. Intriguingly, there is no indication of local structural heterogeneity for His260 and His290, which are known to be crucial in forming Pr substates in the WT.^17^

The assignment of Y176H to a pure Pr–II-like state is consistent with the RR spectra (Figure S5)^16^ and their pH-dependence. In WT Cph1, lowering of the pH from 7.8 to 6.5 leads to the complete conversion from Pr–II to Pr–I as probed by RR spectroscopy,^16^ whereas the RR spectrum of Y176H hardly changes in this pH range, pointing to a single substate (Figure S6). Since in this pH range double protonation of His260 (as the characteristic feature of the Pr–I state) is highly unlikely, we conclude that Y176H remains in a Pr–II-like state in the entire pH range. Furthermore, these findings ensure that the structure derived from the crystals grown at pH 6.9 represents a state with the same protonation pattern as WT Pr–II. Nevertheless, the RR spectrum of Y176H differs from that of WT Pr–II due to the effect of the Tyr → His substitution at position 176 as discussed below.

### Structural Model of the CBP

The crystal structures of the WT and Y176H variants served as the starting point for optimizing a structural model of the CBP using the hybrid QMMM method. In view of the MAS NMR results, we focused on the Pr–II substate with His260 and His290 singly protonated at Nε and Nδ, respectively. For His176 in the Y176H variant, three protonation patterns were considered, namely, double-protonated (HSP) as proposed by NMR spectroscopy and single-protonated at Nε (HSE) or Nδ (HSD). The three geometry-optimized models of Y176H display appreciable changes of the *ZZZssa* PCB structure compared to the WT Pr–II substate (Table S4). In particular, the B–C moiety becomes more planar, while the tilt angle of the A-B methine bridge increases, leading to an out-of-plane distortion of ring A by ∼5° compared to WT Pr–II. This distortion is accompanied by an increase of the C=C and C–C bond lengths of the A-B bridge as reflected by a substantial decrease of the A-B stretching frequency. While these structural changes are largely independent of the protonation pattern of His176, the C-D methine tilting strongly decreases in the order HSD > HSE > HSP. In HSP, ring D is thus rotated by only 27° (with α-facial disposition) compared with 44° in WT Pr–II.

These structural changes of the PCB chromophore are sensitively reflected by the calculated vibrational modes. In line with the previously determined correlation between the C–D dihedral angle and the stretching frequency,^39,40^ the present calculation yielded the highest C–D stretching frequency for the HSP model (1629 cm^–1^). Furthermore, the bond length elongation of the A–B bridge causes a substantial frequency decrease of the corresponding stretching coordinate such that it effectively combines with that of B–C stretching, leading to two modes with frequencies below the C–D stretching, in contrast to the WT Pr–II.^16^

### Validation of the Structural Model by Resonance Raman Spectroscopy

**Figure 4:**
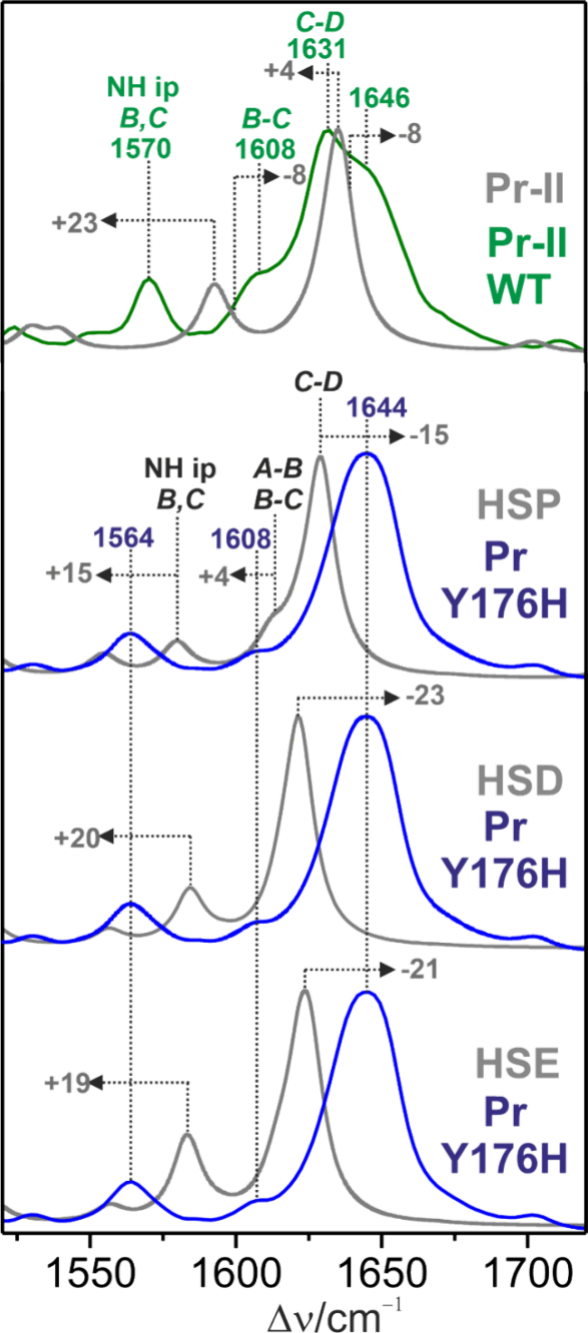
Experimental RR spectra of the Pr–II substate of WT Cph1 (top, green trace) (taken from ref (16)) and the Y176H variant of Cph1 (blue traces). All spectra were measured with 1064 nm excitation at 80 K. The gray traces refer to the calculated spectra based on the structural models of WT Pr–II (ref (16)) and Tyr176 with the single-protonated at Nε and Nδ of His260 and His290, respectively. The additional His176 in Y176H was double-protonated (HSP) or single-protonated at Nε (HSE) or Nδ (HSD).

The assignment of the spectra follows the vibrational analysis of the Pr states of plant phytochromes and Cph1 including isotopically labeled PCB.^16,39−41^ Accordingly and in line with calculations, the most intense RR band is due to the C–D stretching, nearly coinciding with a distinctly weaker C=C stretching of ring D (Figure 4; Table S5).

Unlike the WT protein, resolving these modes is possible in Y176H based on the second derivative presentation (Figure S7 and Table S5), yielding the very intense C–D stretching and the less intense ring D C=C stretching modes at 1650 and 1641 cm^–1^, respectively. In WT Pr–II, the C–D stretching is observed at 1631 cm^–1^ with an intense shoulder at 1646 cm^–1^ on the high-frequency side due to the A–B stretching. On the low frequency side, there are two very weak modes with contributions from the B–C stretching. In Y176H, there is no peak on the high-frequency side of the C–D stretching, implying a substantial downshift of the A–B stretching mode. In fact, these observations can be reconciled with the three protonation models, which all predict a distinct frequency lowering of the A–B stretching coordinate and its effective mixing with the B–C stretching coordinates, leading to two weak bands at 1587 and 1606 cm^–1^ (Figure 4 and Figure S6 and Table S5). The main difference between the three models refers to the C–D stretching mode. The HSP model provides the best description of the high observed frequency of 1644 cm^–1^ and, in addition, reproduces the N–H in-plane bending of rings B and C more accurately (Figure 4). *In toto*, the HSP model of CBP that is the basis for the refinement of the crystallographic structure is consistent with the RR and NMR spectroscopic data.

Interestingly, the equivalent Tyr → His substitution in soybean phyB (*Gm.*phyB Y272H) harboring a PCB chromophore affects the RR spectrum differently than in Cph1 (Figure S7). The C–D stretching mode remains largely unchanged (1640 cm^–1^), and the A–B stretching even shifts from 1647 to 1654 cm^–1^. In spite of this, the spectra of Y272H of phyB are very similar to those of Y176H in Cph1.

### Excited-State Processes

**Figure 5:**
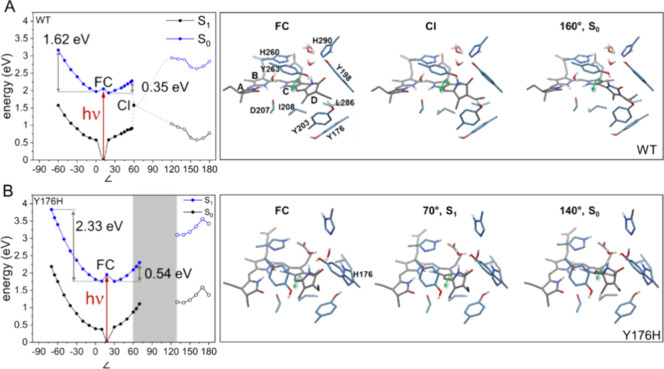
Ground- (S_0_) and excited (S_1_)-state potential energy curves corresponding to the rotation of ring D around the methine bridge between rings C and D (∠ = C14–C15–C16-ND (deg)) for the (A) Cph1 WT and (B) Y176H variant. The red arrow demonstrates the excitation from the S_0_ equilibrium geometry to the Franck–Condon (FC) point, where no significant change in the nuclear coordinate occurs, on the S_1_ potential energy curve. For the S_0_ equilibrium geometry and the FC point, the C14–C15–C16-ND angles were 11.7° and 17.9° for the WT and Y176H Cph1, respectively. From the FC point, clockwise rotation involves proceeding along the curve toward negative dihedral angles (left of the FC point) while counterclockwise rotation involves proceeding along positive dihedral angles (right of the FC point). The full blue circles correspond to the QMMM optimized excited-state (S_1_) geometries, while the open blue circles correspond to vertical excitation energies. All ground-state geometries (black) are optimized. The gray box in panel (B) corresponds to the likely region where the conical intersection (CI) would be located.

Based on the optimized model of the ground-state structure of the CBP, we first studied photoisomerization of PCB by QMMM relaxed scan simulation (Figure 5). Focusing on the C–D methine bridge, we varied the dihedral angle (C14–C15 = C16-ND) around the double bond in both clockwise and counterclockwise directions with a step size of 5°–10° and optimized the geometry in the excited state at each point.

The S_1_ relaxed scans were initiated from the FC point and proceeded in both the clockwise (up to −70°) and counterclockwise (up to 180°) directions. For both the WT and Y176H, a counterclockwise rotation was favored energetically. As the photoisomerization process involves inversion of the bond length alternation, we monitored the changes in the two bonds of the C–D methine bridge. We also monitored the changes in the locations of the amino acid residues surrounding ring D along the reaction coordinate (Figure S8).

In the WT, the QMMM-optimized dihedral angle between the C and D rings was 11.7° for the ground-state minimum geometry (S_0 min_, Figure 5 and Figure S8). In the clockwise direction, the S_1_ geometries converged up to −60° and the S_1_ potential energy curve increased in energy more rapidly than in the counterclockwise direction. In the counterclockwise direction, the calculations converged up to 60°, where the S_1_ and S_0_ energies are degenerate (CI point). Thus, due to the lower barrier, rotation in the counterclockwise direction is more likely. Due to the multireference character of the CI region, we could not optimize points in the S_1_ state beyond 60° and hence continued optimizations in the S_0_ state from 120° to 180°. There were also some interesting changes in the positioning of the amino acid residues along the reaction coordinate that are worth further discussion. The residues Ile208 and Leu286 moved to accommodate the ring D ethyl and ring C methyl groups along the rotation of ring D in the counterclockwise direction. In Figure S8, the ring D ethyl, ring C methyl, Ile208, and Leu286 are represented as van der Waal’s spheres at key geometries along the photoreaction to demonstrate this observation.

The S_0_ optimized geometry for the PCB in Y176H Cph1 included a C14–C15–C16–ND dihedral angle of 17.9°, which was more strongly tilted than that of the WT (11.7°). For Y176H, the corresponding optimized geometries in the S_1_ state were associated with more strongly tilted A–B and C–D methine bridges than in the WT. Counterclockwise rotation is favored, since the barrier was 0.54 eV compared to 2.33 eV for the clockwise rotation. For Y176H, we were unable to see a CI since variation of the S_1_ was limited to 70° due to the multireference character. In contrast to the WT protein, Ile208 and Leu286 do not appear to play a significant role as they interact with the methyl and ethyl side chains of ring D. Rather, in the Y176H protein, the bulky amino acid side chains of Tyr203, Tyr263, and H176 seem to house the ring D in a rather fixed position as the ring D rotation proceeds along the reaction coordinate. Ring D and Tyr203, Tyr263, and H176 are depicted as van der Waal’s spheres in Figure S8 for selected geometries along the reaction path. It is evident that these residues prevent ring D from readily rotating and may be the origin of the increased barrier of 0.54 eV in the counterclockwise rotation relative to the barrier of 0.35 eV in WT Cph1.

**Figure 6:**
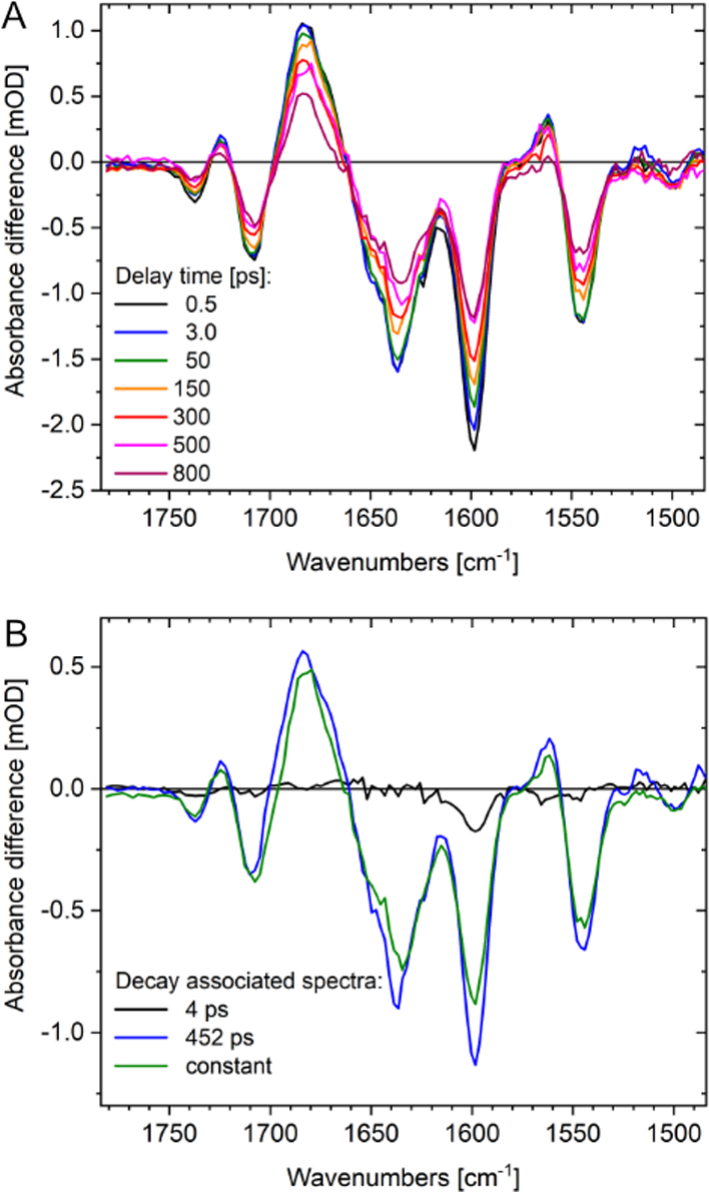
Ultrafast time-resolved vibrational dynamics of Y176H upon excitation at 635 nm in the fingerprint region. (A) Absorbance difference spectra as a function of pump–probe delay time for isotropic polarization. Negative signals indicate the parent Pr ground-state absorption: 1738 cm^–1^ ν(C=O)^A^, 1708 cm^–1^ ν(C=O)^D^, ν(C=C) at 1637, 1599, and 1544 cm^–1^; positive signals indicate the excited-state absorption: 1724 cm^–1^ ν(C=O)^A^*, 1683 cm^–1^ ν(C=O)^D^*, and ν(C=C)* at 1562 and 1515 cm^–1^. (B) Decay-associated spectra for decay times of 4 ps (black line), 452 ps (blue line), and a constant component (green line). The latter show nearly identical spectral features; the 4 ps component exhibits signals only at 1738 and 1596 cm^–1^.

We studied the ultrafast dynamics of the Pr chromophore (D_2_O, pD 7.8) upon excitation at 635 nm. Instantaneously formed positive and negative signals for isotropic polarization reflect the S_1_ and S_0_ state, respectively (Figure 6A).

The dynamics of the difference absorption signals of Y176H are slowed dramatically compared to the WT. The electronic excited state is still clearly visible at 800 ps in Y176H but has already completely decayed at ca. 200 ps in the WT (Figure S9).^42^ No change of the chromophore bands is observed on a time scale of hundreds of picoseconds, indicating a drastically reduced chromophore flexibility in its excited state, compared to WT. For Y176H, the decay of the S_1_ state in the present time window (0–800 ps) follows two exponentials with decay times ca. 4 and 452 ps (Figures S9 and S10), similar to those observed in Agp1 bacteriophytochrome carrying a chemically locked chromophore.^43^ Accordingly, no excited-state decay channel to a photoproduct was detected consistent with the theoretical predictions (vide supra) and in contrast to the WT protein.^18^ The decay-associated spectra (DAS, Figure 6B) display the 452 ps decay and a spectrally very similar constant DAS, indicating a second longer decay time in the ns region. Both spectra exhibit the expected signals of the chromophore dynamics. In contrast to the longer decay times, the 4 ps DAS shows a new feature at 1738 cm^–1^ reflecting protein dynamics.

## Discussion

The present comprehensive characterization of the Y176H variant gives insight into the key structural parameters controlling fluorescence quantum yield as well as the conformational switch associated with the light-induced activation of the WT photoreceptor.

## Chromophore Structure and Fluorescence

The chromophore in the Y176H variant shows *ZZZssa* geometry and is fully protonated, contradicting the notion that Tyr176 is critical for stabilizing the protonation state of the chromophore.^14^ Y176H is also remarkable in showing the largest increase of the fluorescence quantum yield Φ_f_ from 2.4 to 14.5% in a phytochrome brought about by a single substitution. The substitution Tyr263 → Phe results in a Φ_f_ of 8% and together with Tyr176 → His further increases Φ_f_ to 16%.^44^ The fact that the effects of the individual substitutions are not simply additive might imply that they influence the same process, whose effect is limited. The fluorescence of the WT and its increase in Y176H may be rationalized on the basis of a two-state equilibrium between fluorescing and nonfluorescing substates of Pr.^45^ Two substates with different excited-state lifetimes were reported at pH 6 and pH 8.5 with lifetimes of about 30 ps for the Pr–I and lifetimes in the nanosecond range for Pr–II.^18^ Distinct photoisomerization quantum yields were demonstrated for two substates, with about 30 and 3%,^42^ which we assign to Pr–I and Pr–II, respectively. In the WT, the two substates prevailing between pH 6 and 8.5 differ with respect to their excited-state properties since Pr–I is photochemically competent whereas Pr–II is much less active photochemically.^16,18,42^ Correspondingly, Φ_f_ might be higher in Pr–II than in Pr–I, perhaps ca. 5% for Pr–II assuming roughly equal Pr–I and Pr–II occupancies at pH 7.8 where Φ_f_ of the WT was determined.^21,44^ Moreover, it has been shown that the conformational Pr–I/Pr-II heterogeneity has a significant impact of the excited-state processes.^18^ In Y176H, only a Pr–II-like substate is seen, as judged from the protonation pattern of His260 and His290 according to NMR and RR, as well as the fluorescence excitation maximum that is very similar in WT and Y176H.^21^ Hence, the complexity of the excited-state dynamics, as observed for the WT protein,^18^ is reduced, corresponding to a homogeneous ground and excited state. Thus, substitution of Tyr176 by His shifts the equilibrium to Pr–II. The molecular origin may be due to electrostatic interactions with the double-protonated His176 that causes a substantial lowering of the His260 side-chain p*K*_a_. Alternatively, Fischer and Lagarias attributed the high fluorescence of Y176H to the proximity of His176 to the D-ring of PCB, which would hinder double-bond isomerization and stabilize a photochemically inactive, strongly fluorescent state.^24^ This explanation is in line with our ultrafast experiments and would account for the very inefficient photoconversion, but can hardly rationalize the shift of the substate-equilibrium in the ground state.

We thus propose that the structural differences of the chromophore compared to the WT substate Pr–II determine the increase of Φ_f_ in Y176H. This increase seems largely to be due to favoring radiative over nonradiative decay of the electronic excited state since the photochemical activity is already very low in WT Pr–II. In fact, the chromophore of Y176H is homogeneous: The drastically reduced flexibility in the excited state, as shown by ultrafast spectroscopy, hampers thermal energy transfer to the environment and movement along the reaction coordinate including rotation of ring D. This is reflected by the prolonged excited-state lifetime reported here and previously,^24^ and thus leads to increased Φ_f_. However, increased rigidity of the chromophore cannot result from the double protonation of His176 alone; critical structural parameters of the binding pocket are likely to be altered, as well. One of them is most likely the *B*-conformation of the His176/Tyr203 couple (Figure 2) that mimics the Pfr-characteristic of the equivalent residues in bacteriophytochromes.^1^ We propose that the *B*-conformation in conjunction with the Pr-specific *Z*-configuration of the C–D methine bridge reduces the flexibility of the chromophore and thus enhances the fluorescence. This view is supported by the recent finding that also SyB-Cph1, featuring the Pr-like *ZZZssa* PCB configuration together with the Pfr-like α-helical tongue of the PHY domain,^46^ has a rather high Φ_f_ of 12% even as a WT protein, i.e., without any amino acid substitution.^21^

### Protein Conformation and Signaling

The *B*-conformation is associated with numerous other changes in Y176H with respect to the WT, most prominent being the Pfr-typical α-helical structure of the PHY-domain tongue that in the WT Pr state forms a β-sheet (Figure 1). On the other hand, the chromophore remains in the *ZZZssa* configuration, as in WT Pr,^19^ in contrast to the *ZZEssa* configuration in Pfr.^47^ These observations indicate the uncoupling of chromophore and protein structural changes in Y176H. Evidently, Tyr176 is a key residue in translating chromophore isomerization into refolding of the tongue, presumably via the switch of the Tyr176/Tyr203 pair from the *A*- to the *B*-conformation. Similar to Cph1 Y176H, *Dr*BphP Y263F also features a light-independent α-helical PHY-tongue.^48^ Y263F of Cph1 and *Dr*BphP both exhibit increased Φ_f_ compared to the respective WT proteins, but Cph1 Y263F differs as the PHY-tongue adopts a β-sheet structure in Pr.^21,44,49^

A particularly important comparison is with the recently published 3D structure of the homologous variant of a plant type B phytochrome in complex with PIF6.^50^ The structure of *At.*phyB Y276H protein is closely similar to that of the WT in the Pfr state and shows important similarities to Y176H of Cph1, including the Pr-typical ZZZssa configuration of the chromophore and the Pfr-like *B*-conformation of the protein, in particular the α-helical tongue that supports binding to the PIF6 signaling partner. Through this, the observation that Y276H leads to constitutively photomorphogenic (cop) seedling development in darkness can be rationalized by the variant mimicking the function of the WT following photoconversion to Pfr.^26,51^ That conclusion might be analogous for Cph1 and its Y176H variant too. Further support for this interpretation can be derived from the far-reaching similarities between the RR spectra of Y176H and *Gm.*phyB Y272H^27^ (corresponding to *At.*phyB Y276H) (Figure S7). These findings thus underline the value of Cph1 as a model for plant phytochromes.

## Conclusions

In this study, we analyzed the Y176H variant of Cph1 using a combination of structural, spectroscopic, and computational approaches. Crystallography revealed that Y176H adopts a Pfr-like α-helical tongue, while retaining a Pr-like chromophore configuration. MAS NMR and RR spectroscopy identified the protonation patterns of conserved His260 and His290 in the CBP. It was found to be homogeneous and the same as in the Pr–II conformational substate, eliminating the heterogeneity observed in the WT. Furthermore, the joint NMR, RR, and QMMM analyses demonstrated a double-protonated (cationic) His176. This is most likely an essential parameter for the increased fluorescence yield of Y176H that is reflected by the increased photoisomerization barrier and prolonged excited-state lifetime shown by QMMM simulation and ultrafast spectroscopy. Moreover, the cationic His176 decouples chromophore isomerization from the structural transition of the tongue. Thus, Y176H represents a mixture of a Pr-like chromophore configuration with a Pfr-like protein structure. Altogether, this work advances our understanding of the molecular properties of phytochromes in two aspects. First, it potentially offers a rationale for designing improved fluorescent variants for bioimaging applications. Second, it provides mechanistic insight into the structural communication between the chromophore and those entities of the protein that are essential for biological functions.

## Supporting Information Available

Details about experimental and computational methods, further structural and spectroscopic data (PDF)

## Accession Codes

Coordinates for the Cph1 Y176H variant have been deposited in the Protein Data Bank as entry 8RVX.

## Dedication

This paper is dedicated to the memory of Prof. Dr. Ulrike Alexiev, Freie Universität Berlin, who passed away on December 29, 2023.
