# Supplementary material for: Integrated Study of Fluorescence Enhancement in the Y176H Variant of Cyanobacterial Phytochrome Cph1
Source: Biochemistry. 2025 Feb 27;64(6):1348–58. doi: 10.1021/acs.biochem.4c00687 (PMC11924222; doi:10.1021/acs.biochem.4c00687)
Supplement: Supplementary file 1 — bi4c00687_si_001.pdf [file bi4c00687_si_001.pdf]

Supporting Information

Integrated study of fluorescence enhancement in the Y176H variant of cyanobacterial phytochrome Cph1

Soshichiro Nagano[1,2*], Chen Song[3], Valentin Rohr[3], Megan J. Mackintosh[4], Oanh Tu Hoang[5], Anastasia Kraskov[5], Yang Yang[6], Jon Hughes[1,6], Karsten Heyne[6], Maria-Andrea Mroginski[5], Igor Schapiro[4,7], Peter Hildebrandt[5,*]

[1] Institute for Plant Physiology, Justus Liebig University, Senckenbergstr. 3, D-35390 Giessen, Germany

[2] Present address: Department of Biomolecular Mechanisms, Max Planck Institute for Medical Research, Jahnstr. 29, D-69120 Heidelberg, Germany

[3] Institute for Analytical Chemistry, University of Leipzig, Johannisallee 29, D-04103 Leipzig, Germany

[4] Fritz Haber Center for Molecular Dynamics, Institute of Chemistry, Hebrew University of Jerusalem, Jerusalem 91904, Israel.

[5] Technical University of Berlin, Institute for Chemistry, Str. des 17. Juni 135, D-10623 Berlin, Germany

[6] Department of Physics, Free University of Berlin, Arnimallee 14, D-14195, Berlin, Germany

[7] Present address: Department of Physics, Technische Universität Dortmund, D-44227 Dortmund, and Research Center Chemical Sciences and Sustainability, University Alliance Ruhr, D-44801 Bochum, Germany

*Corresponding authors:

S.N.: soshichiro.nagano@mr.mpg.de

P.H.: peter.hildebrandt@tu-berlin.de

1. Detailed descriptions of methods and results

1.1. MAS-NMR spectroscopy

Experimental details. Optimized ^{1}H, ^{13}C, and ^{15}N $\pi/2$ pulse lengths were 2.3, 3.5, and 3.6 μs, respectively. For ^{13}C and ^{15}N CP experiments, ^{13}C and ^{15}N transverse magnetization created by ramped CP (100–70%) was transferred from ^{1}H with an optimal contact time of 2 ms. The r.f. lock field of 54.7 and 39.3 kHz was applied on ^{13}C and ^{15}N, respectively, fulfilling the Hartmann–Hahn condition. During the acquisition, a swept-frequency two-pulse phase modulation heteronuclear decoupling (SW$_f$-TPPM) at a ^{1}H r.f. field of 113.2 kHz was used for ^{1}H decoupling.[1] For the 1D ^{15}N CP spectrum, 96256 scans were accumulated with a relaxation delay time of 2.5 s. A line broadening of 20 Hz and zero-filling to 4096 points were used prior to Fourier transformation.

2D ^{13}C–^{13}C DARR spectra were acquired with a CP contact time of 2 ms and the two proton mixing times of 5 and 50 ms. In total, 114 t_1-increments were accumulated with 2176 scans in each indirect slice and a relaxation delay of 2.5 s. The ^{1}H–^{13}C dipolar interaction has been recovered by continuous wave irradiation at r.f. field of 19.1 kHz satisfying the $n = 1$ rotary-resonance condition.[2] During the acquisition a SW$_f$-TPPM heteronuclear decoupling was applied. A 45° shifted squared sine-bell window function (SSB = 2) and zero-filling to 2048 points was applied to the indirect dimension. A 90° shifted squared sine-bell window function (SSB = 2) was applied in the direct dimension and further zero-filled to 4096 points. 2D ^{1}H–^{13}C/^{15}N HETCOR data were collected using frequency-switched Lee–Goldburg ^{1}H homonuclear dipolar decoupling.[3] The ^{1}H chemical shift scaling factor was determined experimentally to be around 0.57.[4] Both HETCOR experiments were acquired with a CP contact time of 2 ms, 60 increments in the indirect dimension and 3840 and 3856 scans for each increment in the ^{13}C and ^{15}N channels, respectively, with a relaxation delay of 2.0 s. In both cases, a 90° shifted squared sine-bell window function (SSB = 4) and zero-filling to 1024 points was applied to the indirect dimension. A 90° shifted squared sine-bell window function (SSB = 2) was applied in the direct dimension and further zero-

filled to 4096 points. ^{13}C chemical shifts were externally referenced to the α-glycine C(O)O$^-$ resonance at 176.04 ppm on the TMS scale and ^{15}N chemical shifts were referenced to the Nα resonance of histidine·HCl at 49.04 ppm on the liquid NH_3 scale. The data was processed with Bruker Topspin 4.0.1 and further analyzed with MestReNova 14.1.0 (Mestrelab Research, Santiago de Compostella, Spain).

Detailed description of results. We initially performed two DARR experiments with different ^1H mixing times to observe direct ^{13}C–^{13}C connectivities (5 ms) and medium-range carbon atom pairs (50 ms) such as C8/C10 and C15/C17^1 (Figure S2). Moreover, the intramolecular distances of correlations arising from C1/C3^1, C2/C5, and C9/C7^1 are of 3.6–3.7 Å (as extracted from the 3D structure of Cph1-Y176H mutant, PDB code 8RVX) which roughly represent the effective range for the detection of weakly dipolar-coupled ^{13}C spins at the mixing time of 50 ms. Strikingly, almost all PCB carbon atoms showed a single DARR correlation network, with the exceptions the B-ring propionate carboxylate group (C8^3), for which two sets of ^{13}C chemical shifts were identified with resonance separations of ~1.5 ppm (Figure S4). By contrast, the widespread resonance splitting was observed in the WT protein for the A-ring carbons (C1, C2, C3, and C3^2), the B-ring propionate sidechain (C8–C8^3), and the two D-ring methyl groups (C17^1 and C18^2) in the corresponding Pr state (Table S3), implicating multiple conformational states attained by the chromophore.

Following the complete set of ^{13}C assignments, we next acquired ^1H–^{13}C correlation spectra with the two CP contact times of 300 and 800 μs (Figure 3B). By choosing a contact time of 300 μs, the spectrum is mostly for one- and two-bond intramolecular correlations of the chromophore itself, e.g., C12^1/C11–H12^1. However, with a longer contact time of 800 μs, a set of interfacial contacts between the chromophore carbon atoms and the protons bound to proximal residues in the binding pocket were resolved. According to the Cph1-Y176H structure, the imidazole ring of H176 is closely associated with the D-ring ethyl sidechain (Figure 2). This disposition is confirmed by the two correlation peaks involving its imidazole protons (Hε1 and Hε2 with δ^H of 7.0 and 9.2 ppm, respectively) and C18^2.

According to the crystal structure, the sidechain of H290, highly conserved in all known phytochromes, is hydrogen-bonded to the D-ring carbonyl group of the chromophore (C19) via its imidazole Hε2 proton. We therefore assigned this strong interaction to an intense correlation in the most downfield region at δ^H = 11.2 ppm. C19 also received the ^{1}H magnetization from the imidazole Hε1 proton at δ^H = 7.2 ppm. Moreover, H260, fixing the coplanar B- and C-rings from their α-face, exhibits two correlations involving C11 and C12^1 of the ring C with its imidazole Hε2 proton (δ^H = 10.4 ppm) at a distance of ~3.9 Å (C11/C12^1···Nε2). The appearance of these two correlations suggests that the imidazole disposition of H260 in the mutant is nearly identical to that of the WT, for which a distance of ~3.8 Å was predicted.[5] Intriguingly, the correlation data do not reveal any local structural heterogeneity of the protein environment around the PCB chromophore, particularly regarding His260 and His290 which are known to be crucial in forming Pr isoforms in the WT species, distinguished by the protonation states, rotameric structures of their imidazole rings and the associated hydrogen bonds to the chromophore.[5]

The ^{15}N MAS NMR spectrum of the Y176H mutant displays far-reaching similarities to that of the WT.[6,7] The unequivocal ^{15}N assignment of tetrapyrrole nitrogens in the WT achieved by dynamic nuclear polarization (DNP) enhanced MAS NMR[6] thus forms the basis for assignment of the mutant spectrum (Figure S3). Four signals can directly be quantified by a Voigt deconvolution, yielding a ratio of 0.8:1.0:1.1:1.0 with their maxima below 161.5 ppm and a δ^N dispersion of ~33.9 ppm (as $\delta^{N21} - \delta^{N24}$). This demonstrates that the chromophore remains fully protonated as in the WT protein, contradicting the proposal that Y176 is critical for stabilization of the protonation state of chromophore.[8] Moreover, the Y176H mutation enlarges the δ^N dispersion of the four pyrrole nitrogens by 7.2 ppm (from ~26.7 ppm as WT),[6] implicating a shorter conjugated system of chromophore. This may reflect, for example, an increased out-of-plane torsion of ring A or D or both. However, in view of the QMMM geometry optimization and the RR data, a stronger tilted A-ring but less tilted D-ring conjugation appears to be the most plausible PCB geometry.

Based on the ^{15}N chemical shifts of pyrrole nitrogens (Figure S3A), 2D ^{1}H–^{15}N correlation spectrum allowed us to assign the NH protons of pyrrole nitrogens of rings A–C (N21–N23) (Figure S3B) through the correlation with their directly bonded nitrogens, resonating at 161.5, 155.3 and 145.4 ppm, respectively. Compared with the WT protein, the Tyr176→His substitution produced only subtle δ^{H} changes (≤ 0.3 ppm) at the NH protons of rings A–C (H21–H23). Unlike the PCB carbons and nitrogens, δ^{H} values of NH protons are less sensitive to the electronic and geometric structures of the chromophore but determined mainly by their interactions with the residues nearby. It is thus clear that the key protein–chromophore interactions involving the pyrrole nitrogens of rings A–C (N21–N23) are well conserved in the mutant, consistent with the crystal structure. However, the ^{1}H correlation of N24 (ring D) is less resolved due to the partial overlap with the amide resonances originated from protein backbone in natural abundance and thus not included in Figure S3.

The prominent δ^{C} changes in Y176H are not confined to certain chromophore region such as rings C and D, but also detected at C4 and C6 atoms associated with the C5-methine bridge (Figure 3, Figure S2). These changes implicate twisting and/or bending motions of ring A (relative to the ring B). Unlike the conformational rearrangement of the two outer rings, rotation around the C10-methine carbon is likely to be minor. This hypothesis is supported by $|\delta^{C}|$ changes of C9–C11 bridging the two inner rings that are shifted ≤ 2.3 ppm (Figure S2). The chemical shifts for these pyrrolic carbons of the C10-methine bridge are largely determined by the conjugation of the two inner rings and methine bridge configuration. Retention of configuration of C10 is consistent with the maintenance of protein–chromophore interactions at the rings B and C. Moreover, the coplanar B–C plane in this mutant is somewhat more tightly fixed than in WT, considering, for example imidazole ring of H260 lying in its α face is ~0.3 Å (in average for each ring atom) more closer to the plane.

The observed δ^{C} changing pattern of the π-conjugated C4–C19 system indicates that the entire conjugation chain undergoes significant change (Figure S4). On the left side of ring C, all pyrrolic

carbons show decreased electron density (blue) whereas an increased electron density is associated with most carbons on the right side (red) with the exceptions of C12, C15 and C17 (less than 1.4 ppm). Such an overall changing pattern rules out the possibility that the chromophore in the mutant mimics the conjugated system and geometry of the WT chromophore in any of the intermediates,[9] and also implies a perturbation of the typical bond order (single or double bond character) within the conjugated chain.[7,10] Moreover, a distribution shift of positive charge mainly delocalized between rings B and C of the chromophore may take place associated with the change of bond alternation.[10]

1.2. Ultrafast spectroscopy

We measured the absorption differences for parallel and perpendicular polarization (A_{par} and A_{per}, respectively) with respect to the pump pulse polarization. The signals for isotropic polarization A_{iso} were taken from $A_{iso} = (A_{par} + 2 \cdot A_{per})/3$. The pulse energy of the pump and probe was 0.6 μJ and 10 nJ, respectively. Polarization-resolved fs VIS-pump IR-probe experiments were performed on Y176H to measure the relative angle between the electronic transition dipole moment (tdm) of the electronic $S_0 \rightarrow S_1$ transition and the vibrational transition dipole moment (vtdm) of individual vibrational modes (Figure S9). Polarization-resolved DAS provides the relative angles for the C=O stretching modes of ring *A* (ca. 55°) and *D* (ca. 43°) upon electronic transition. We observe negligible changes on a time-scale of hundreds of picoseconds. As no rotation of ring D in S_1 is seen, the conical intersection (CI) cannot be reached and photoisomerization cannot take place. Similar observations were made on locked Agp1 bacteriophytochrome[11] and contrast with data from WT Cph1.[12] For delay times on a nanosecond time-scale, reflected by the constant DAS (Figure S10), the strengths of parallel and perpendicular signals are nearly identical, implying rotational diffusion and/or increased flexibility of the entire chromophore within the protein binding pocket. Direct comparison of the measured C=O orientations between Y176H and WT is not reasonable, since in contrast to Y176H, the WT experiments were performed with $^{13}C/^{15}N$

labelled protein. Hence, protein contributions in Y176H can contribute to the experimentally determined orientation of absorption bands of the C=O stretching mode masking their true orientation. This possible interference may be the main origin for the discrepancy between peak position of the C=O stretching mode in the transient IR difference signal and the absolute RR spectrum (Figure S5), which in addition was measured at 90 K.

The fastest dynamic component in Y176H of 4 ps is very small and shows an absorption pattern not following chromophore signatures as present in the 452 ps and constant component. This points to protein contributions altered due to the chromophore excitation, probably induced by electric field changes around the chromophore due to electron density shifts in the electronic excited state S_1. The band around 1738 cm^{-1} could arise due to changes of a carbonyl or an arginine mode.

1.3. Extended methods for molecular dynamics simulations and quantum mechanical calculations

For MD simulations, the protein models were solvated in a rectangular TIP3P water box and Na^+ ions were added to neutralize the systems using the *tleap* program in AMBER16. The parameters for the PCB chromophore were derived in a previous study[13] and used here. The SHAKE algorithm was used for the bonds involving hydrogen atoms. The non-bonded interaction cut-off was 12.0 Å and the Particle Mesh Ewald algorithm was used to treat long-range interactions. The Langevin thermostat with a collision frequency of 1 ps^{-1} was used to control the temperature. The initial MD simulation was a minimization in 20000 steps with restraints of 420 kJ $mol^{-1}Å^{-2}$ on all atoms of the protein and PCB. Then the models were heated from 100 K to 300 K in 1 ns with the restraints on the protein and PCB in NVT conditions. Subsequently, the systems were gradually equilibrated for 5 ns in NPT conditions with a restraint weight of 100 kcal $mol^{-1}Å^{-2}$. This was followed by further equilibrations with a weaker restraint weight of 10 kcal $mol^{-1}Å^{-2}$ for an additional 5 ns. The models were then optimized with restraints on the protein backbone alone for 10000 steps. This was followed by a gradual reduction in the restraint weight from

10 to 1 kcal $mol^{-1}Å^{-2}$ and then 0.1 $mol^{-1}Å^{-2}$ where each simulation was 5 ns and the protein backbone atoms remained constrained. Next, all constraints on the protein were lifted for a 50 ns unrestrained simulation. Finally, the MD production run of 1000 ns (1 μs) was carried out.

For the excited state relaxed scan, a geometry was selected from each production run for each model. The WT and the Y176H model contained HIE260/HID290 and HIE260/HID290/HIE176, respectively. Prior to the relaxed scan calculations, each snapshot was optimized using the hybrid the QM/MM method. Optimization was carried out at the density functional theory (DFT) level with the BP86 functional and the cc-pVDZ basis set with dispersion corrections and Becke-Johnson damping included. The MM region was treated with the AMBER ff14sb force field. In line with previous studies.[13,14] the QM region contained a truncated PCB chromophore of 66 atoms including 3 link atoms. The chromophore was truncated at the ring *A* side chain which formed a C-C bond to the cysteine. Another QM-MM boundary was placed at the propionate side chains between the sp3 carbons. A QM region of this size has been shown to be suitable for excited state calculations involving phytochromes because it does not truncate the conjugated system. Any residue which had an atom within 5 Å of the chromophore was allowed to freely move throughout the optimization. The QM/MM calculations were carried out with ChemShell interfaced with Orca and the L-BFGS approximation was employed in the internal DL-FIND module of Chemshell for the optimization while the DL-POLY module was employed for the MM portion. The QM/MM optimized models were used as the starting point for the S_1 excited state surface scan along the isomerization of the double bond between ring C and D. ChemShell interfaced with Turbomole 7.0 was used for the excited state calculations. The ChemShell DL-FIND module was used for the optimization and the adiabatic excitation energies were computed with the algebraic diagrammatic construction to the second order (ADC(2)) with the cc-pVDZ basis set in Turbomole 7.0. The QM region contained truncated PCB with 66 atoms and MM region contained the protein residues with the water molecules, as described above. All residues and water molecules with an

atom within 5 Å of PCB were unfrozen throughout the optimization. Notably, the model systems contained nearly 100,000 atoms with the TIP3P water boxes and despite several attempts, this model size was unfeasible for the ChemShell/Turbomole calculations for the S_1 excited state relaxed scan. To overcome this hurdle, a 20 Å sphere of water molecules around the PCB was retained for the relaxed scan calculations which brought the model sizes to ~40,000 atoms. The dihedral angle between ring C and D (∠C14-C15=C16-N_{RingD}) was systematically increased by 10° in both clockwise and counter-clockwise directions until the limit of the single-reference ADC(2) method was reached (as apparent from calculated negative excitation energies).

2. Supporting figures and tables

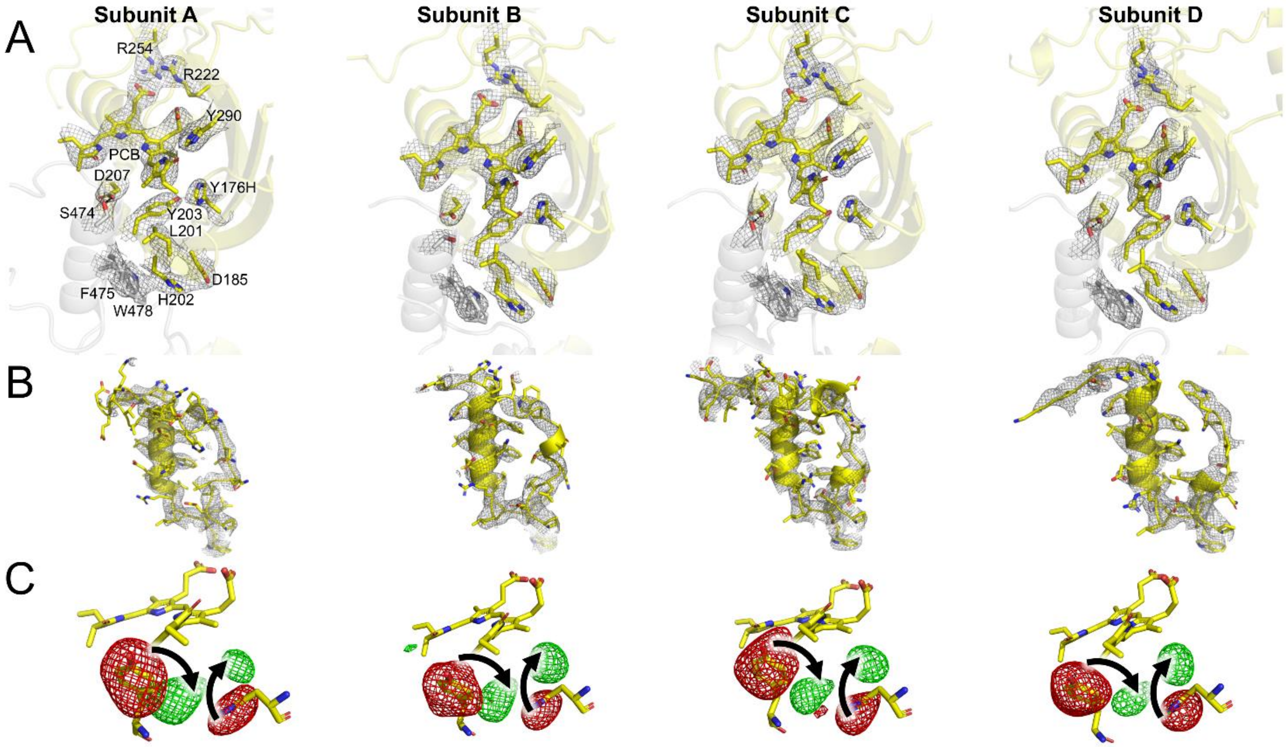

Figure S1. Structural details of the four subunits of the Cph1 Y176H crystal structure. A, The chromophore and adjacent residues involved in signal transductions. Electron density map (2Fo – Fc) contoured at 1.0 rmsd. B, α-helices at the PHY-domain tongues (residues 443-486). Electron density maps (2Fo – Fc, 1.0 rmsd contour). C, Tyrosine dyad in the *B*-conformation. Refined Y176H structure with the tyrosine dyad in the WT-like *A*-conformation results in difference signals (Fo - Fc, 3.5 rmsd contour level) and compared to the *B*-conformation (indicated by black arrows).

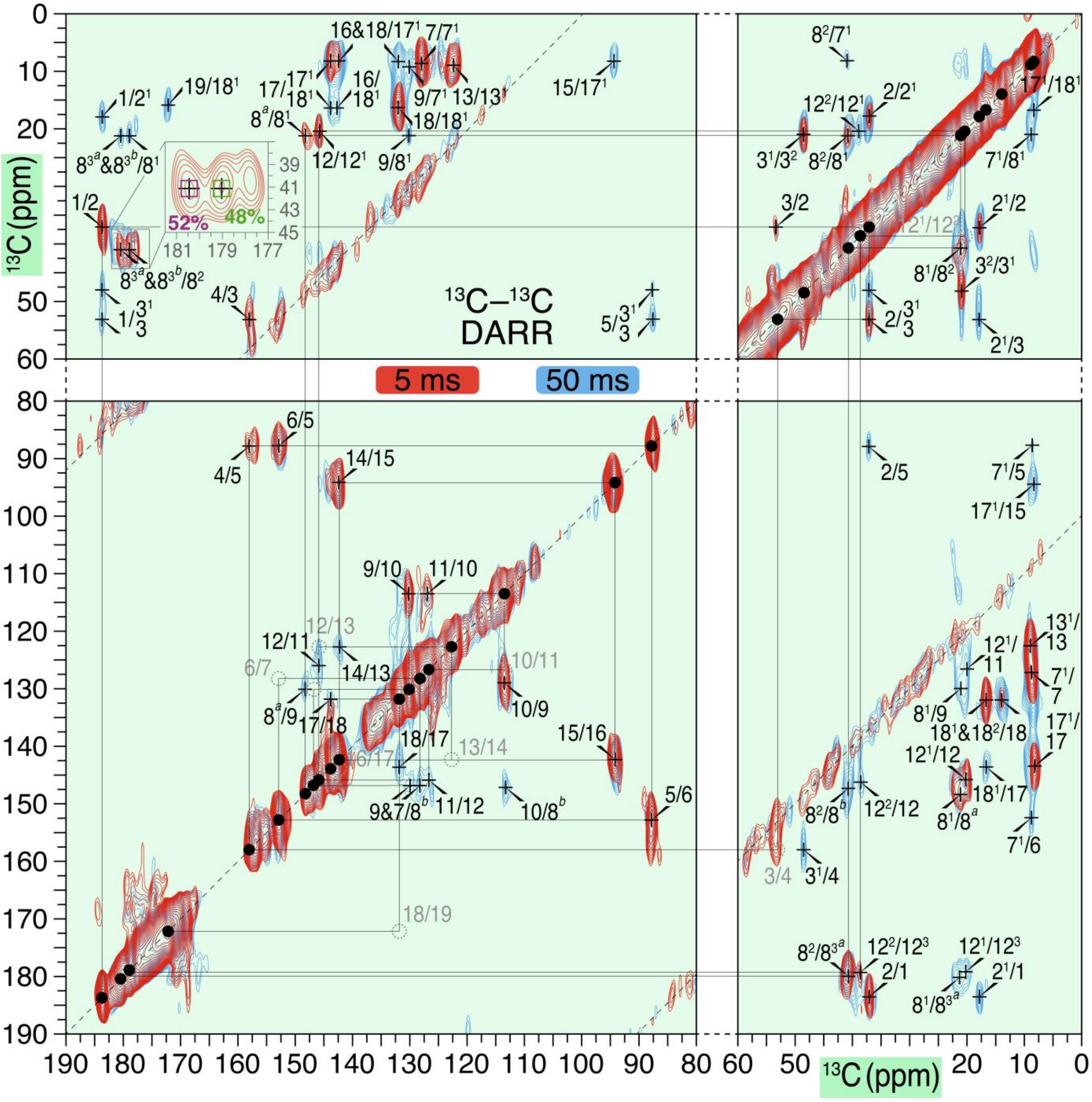

Figure S2. Enlarged contour plots of the DARR spectra shown in Figure 3 with complete chromophore assignments. All carbon pairs of the PCB chromophore are indicated by arrows and labeled. The gray lines indicate sequences of directly-bonded carbon pairs. Enlarged view of the $C8^3/C8^2$ signal doubling is inset. The numbers given correspond to their relative intensities after integration.

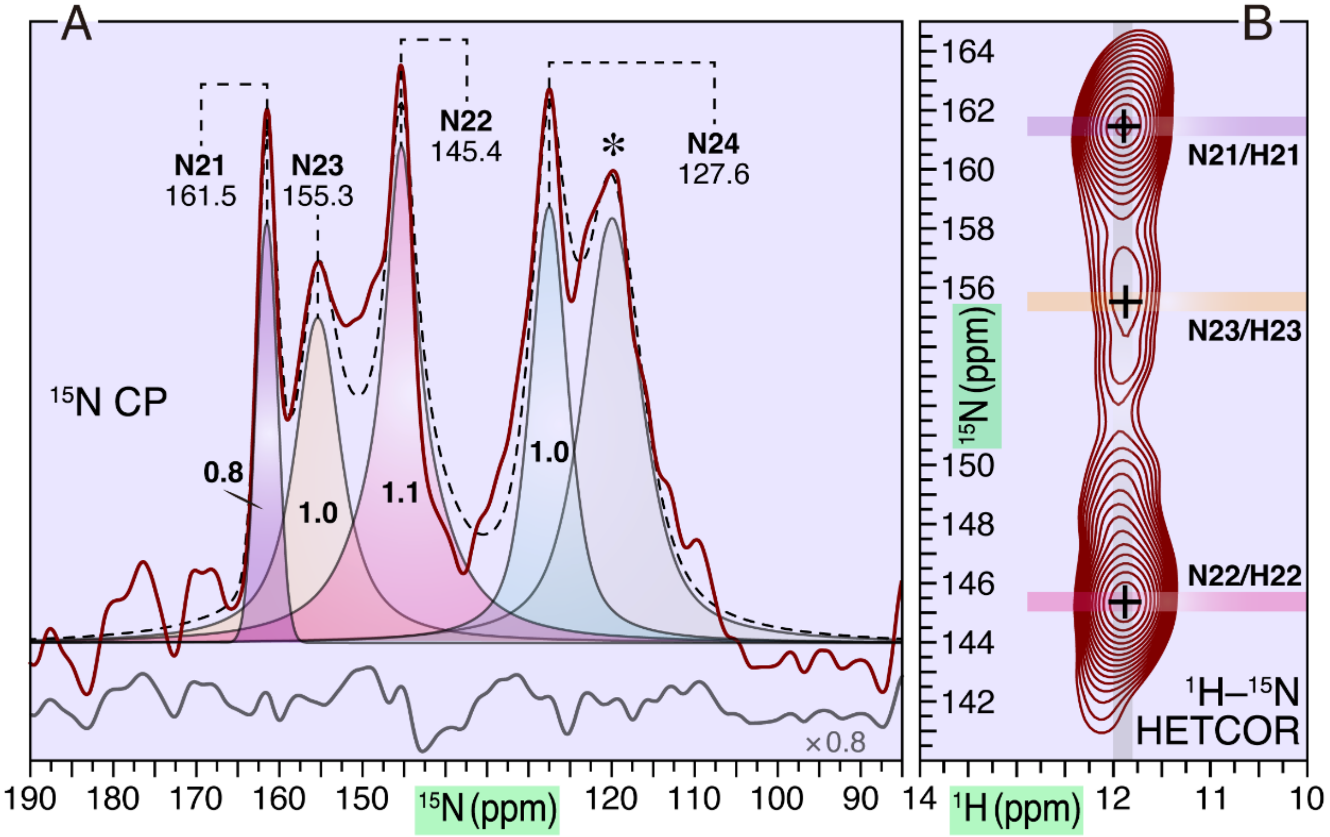

Figure S3. ^{15}N MAS NMR data of *u*-[^{13}C, ^{15}N]-PCB-Cph1 Y176H in the Pr state. (A) Voigt deconvolution of the ^{15}N CP/MAS spectrum of the Y176H mutant. A Voigt function with a mixed Lorentzian–Gaussian ratio of ~1:1 was applied to fit the experimental curves (colored brown). Fitted spectrum is shown in black (dotted line). Residual errors of the Voigt deconvolution are given at the bottom (colored gray). δ^N of individual Voigt fits are labeled. Relative area (shaded as purple, pale yellow, pink, and pale cyan for N21, N23, N22, and N24, respectively) under each Voigt fit was calculated as setting N24 signal to unit and labeled by black numbers. Signals arising from backbone amide are denoted by asterisks and shaded as light gray. (B) Contour plot of 2D ^{1}H–^{15}N HETCOR spectrum. Only the characteristic spectral regions 10–14 (^{1}H dimension) and 140–164 ppm (^{15}N dimension) are shown.

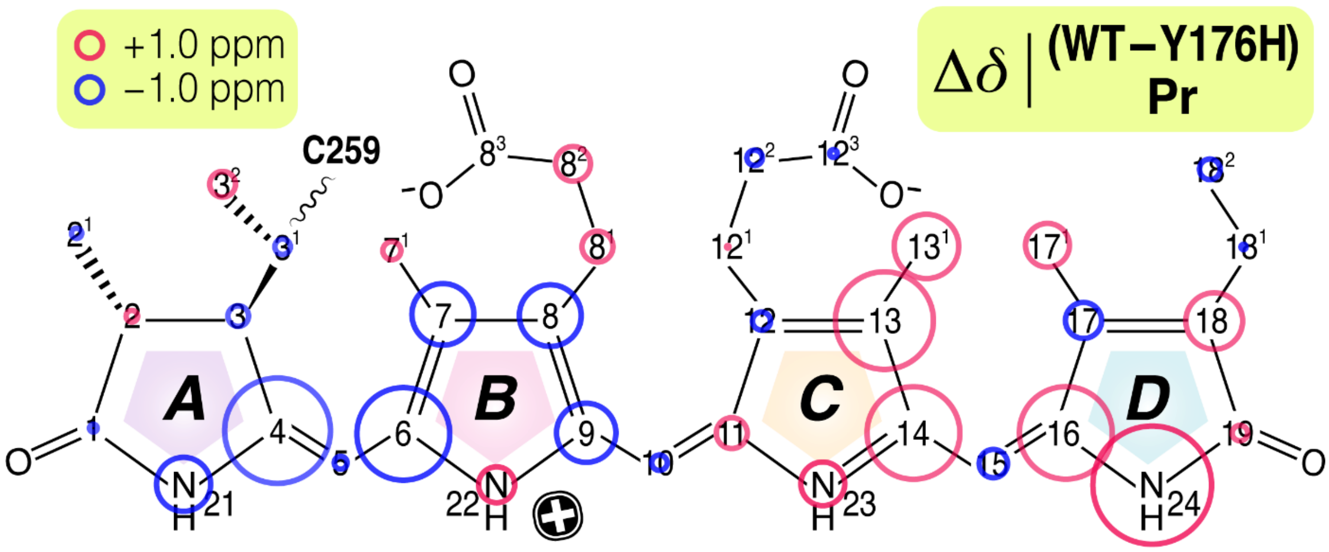

Figure S4. Schematic of the changes in ^{13}C and ^{15}N chemical shifts of the chromophore in WT and its Y176H mutant. The corresponding changes are represented as red and blue circles for down- and upfield shifts, respectively. The size of the circles is proportional to the chemical shift differences as WT minus Y176H, see also Table S3.

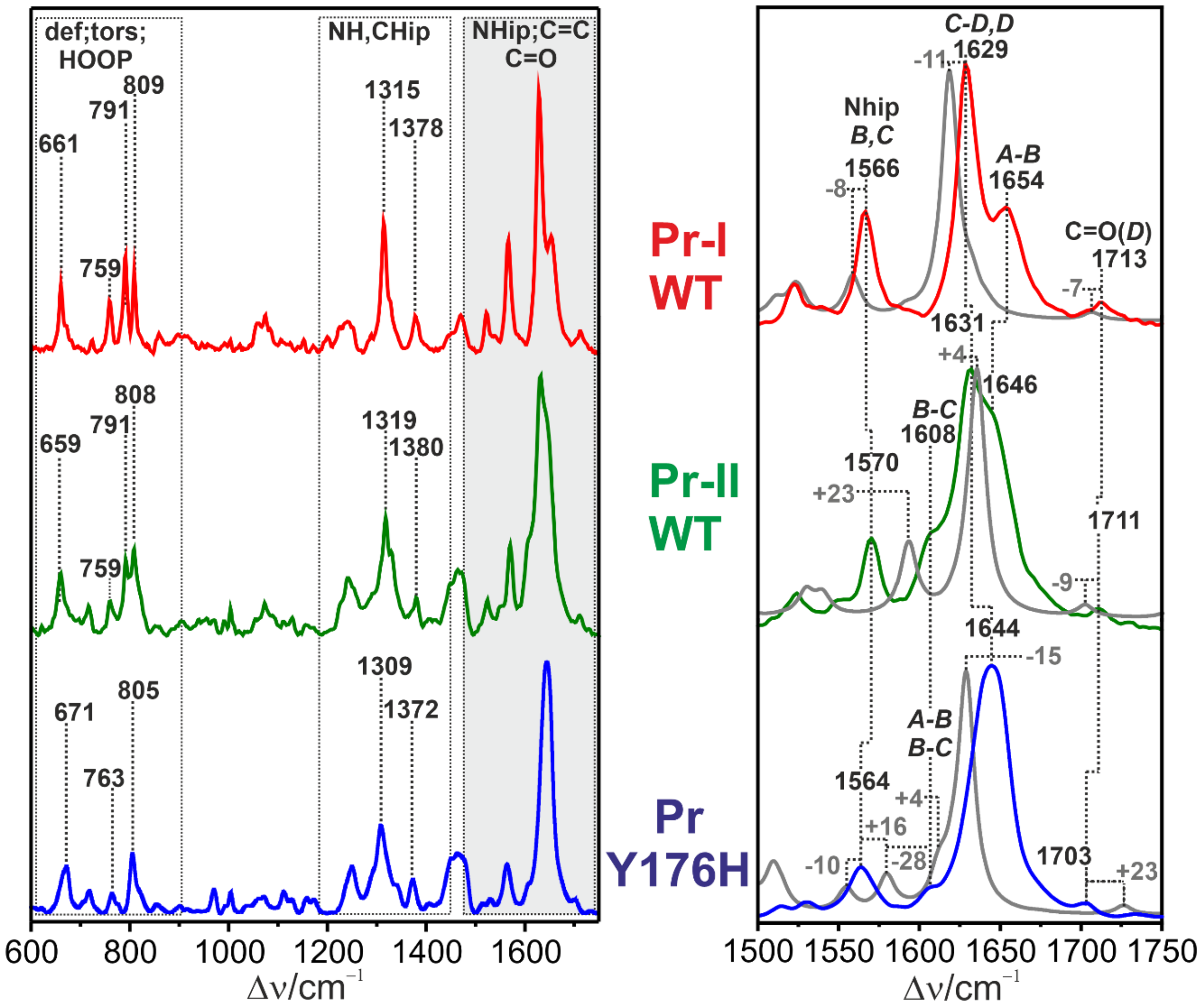

Figure S5. RR spectra of Y176H and the two sub-states Pr-I and Pr-II of WT Cph1, measured at 90 K with 1064 nm excitation. The left and right panel show an overview of the spectra and an expanded view of the C=C stretching region, respectively. Spectra of WT Pr-I and Pr-II were taken from previous work.[15] The main difference between Pr-I and Pr-II refers to the A-B stretching mode, which is distinctly lower in frequency in Pr-II (1646 cm^{-1}) than in Pr-I (1654 cm^{-1}) and thus more similar to Y176H in which the A-B stretching is even further downshifted. Note that the most intense peak in the spectra is due to two closely spaced bands of very strong and medium Raman activity that originate from the C-D and ring D C=C stretching, respectively (see Figure S7). The grey traces refer to the calculated spectra based on the structural models.

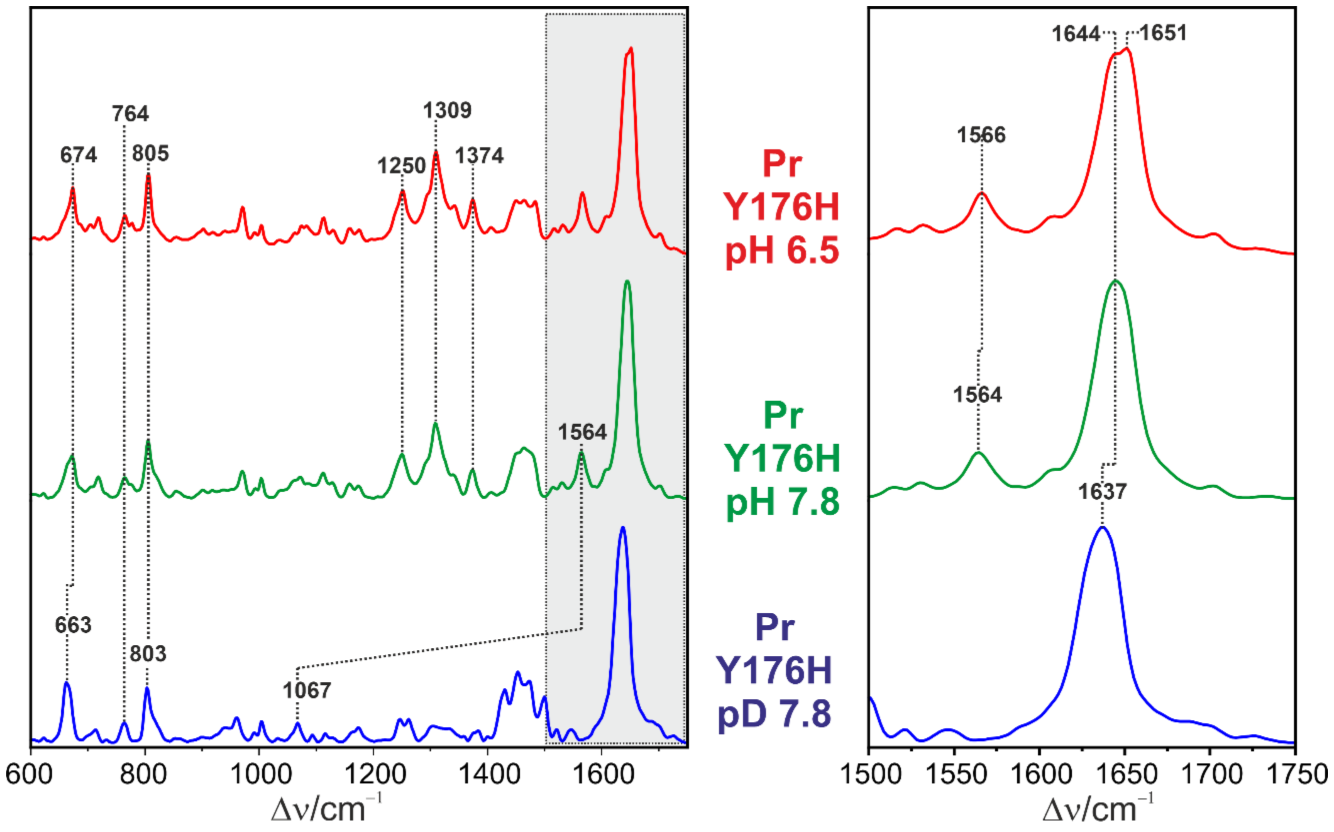

Figure S6. RR spectra of Y176H measured at 90 K with 1064 nm excitation. Red and green traces refer to the substates different pH values as indicated. The blue trace is the spectrum at pD 7.8. The left and right panel show an overview of the spectra and an expanded view of the C=C stretching region, respectively. Upon H/D exchange, the NH ip bending of rings B and C shifts from 1564 to 1067 cm^{-1}. The good agreement of the spectra at pH 6.5 and 7.8 confirms that in this pH range there is no change of the protonation pattern of His260 and His290 which in the WT protein are associated with the Pr-I/Pr-II equilibrium. This finding also implies that the protonation state of the protein (including His176) is the same at pH 6.9 used for crystallization and pH 7.8 used for all spectroscopic measurements.

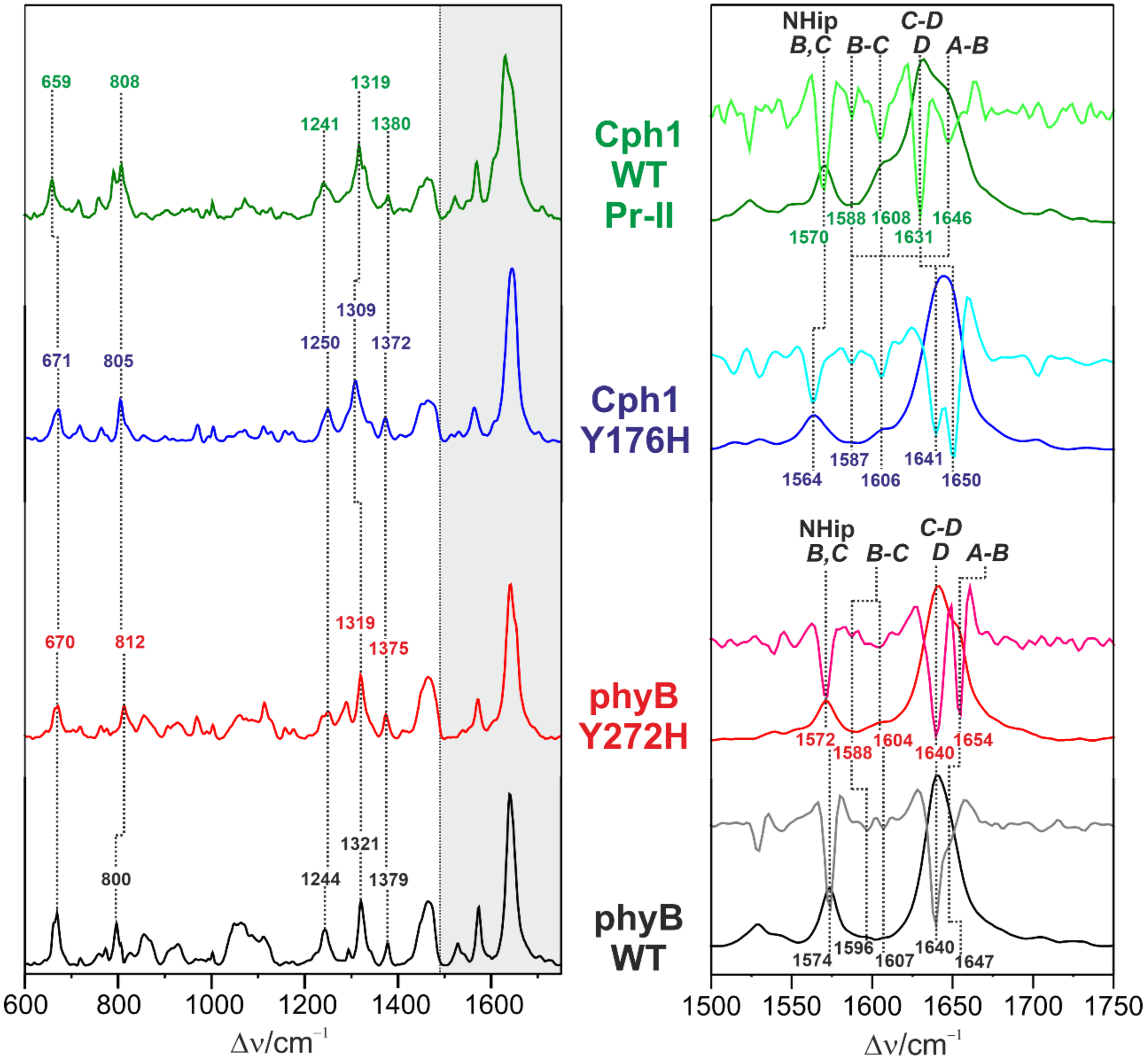

Figure S7. RR spectra of Cph1 and phyB measured at 90 K with 1064 nm excitation. Blue and green traces are the Y176H and WT spectra of Cph1, respectively. Black and red traces refer to the Pr state of the WT phyB and its Y272H variant, respectively. WT spectra of Cph1 and phyB were taken from previous studies.[15,16] The left panel shows an overview of the spectra and the right panel displays an expanded view of the C=C stretching region, including the second derivatives. The latter allows identifying band components in overlapping peaks, which is particularly important for separating the closely spaced C-D stretching and ring D C=C (D) stretching modes.

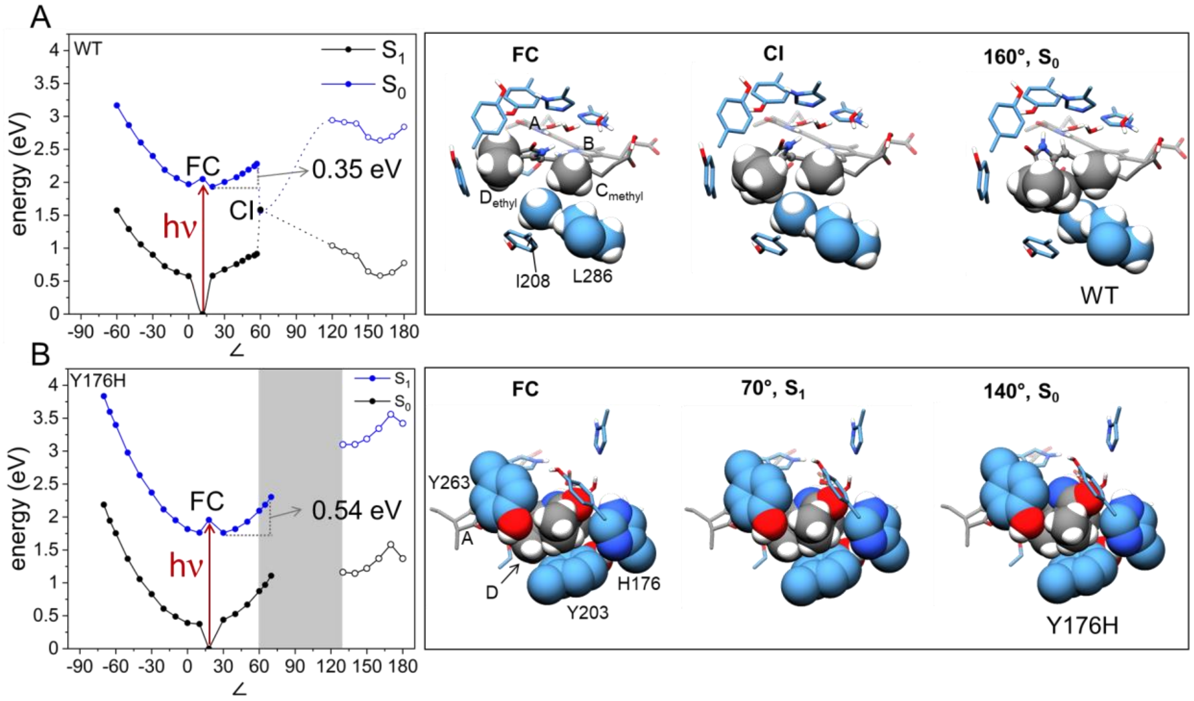

Figure S8. Ground (S_0) and excited (S_1) state potential energy curves corresponding to the rotation of ring D around the methine bridge between rings C and D (∠ = C_{14}-C_{15}-C_{16}-N_D (°)) for the Cph1 wild type (A, left) and Y176H variant (B, left). The full blue circles correspond to the QM/MM optimized excited state geometries while the open blue circles correspond to vertical excitation energies. All ground state geometries (black) are optimized. The gray box in panel B corresponds to the likely region where the conical intersection (CI) would be located. The right hand side of panel A are selected geometries along the reaction pathway with the ethyl and methyl groups of ring D and ring C, respectively, and Ile208 and Leu286 depicted with van der Waals spheres. The right hand side of panel B are selected geometries along the reaction pathway with ring D and His176, Tyr203, and Tyr263 depicted with van der Waals spheres.

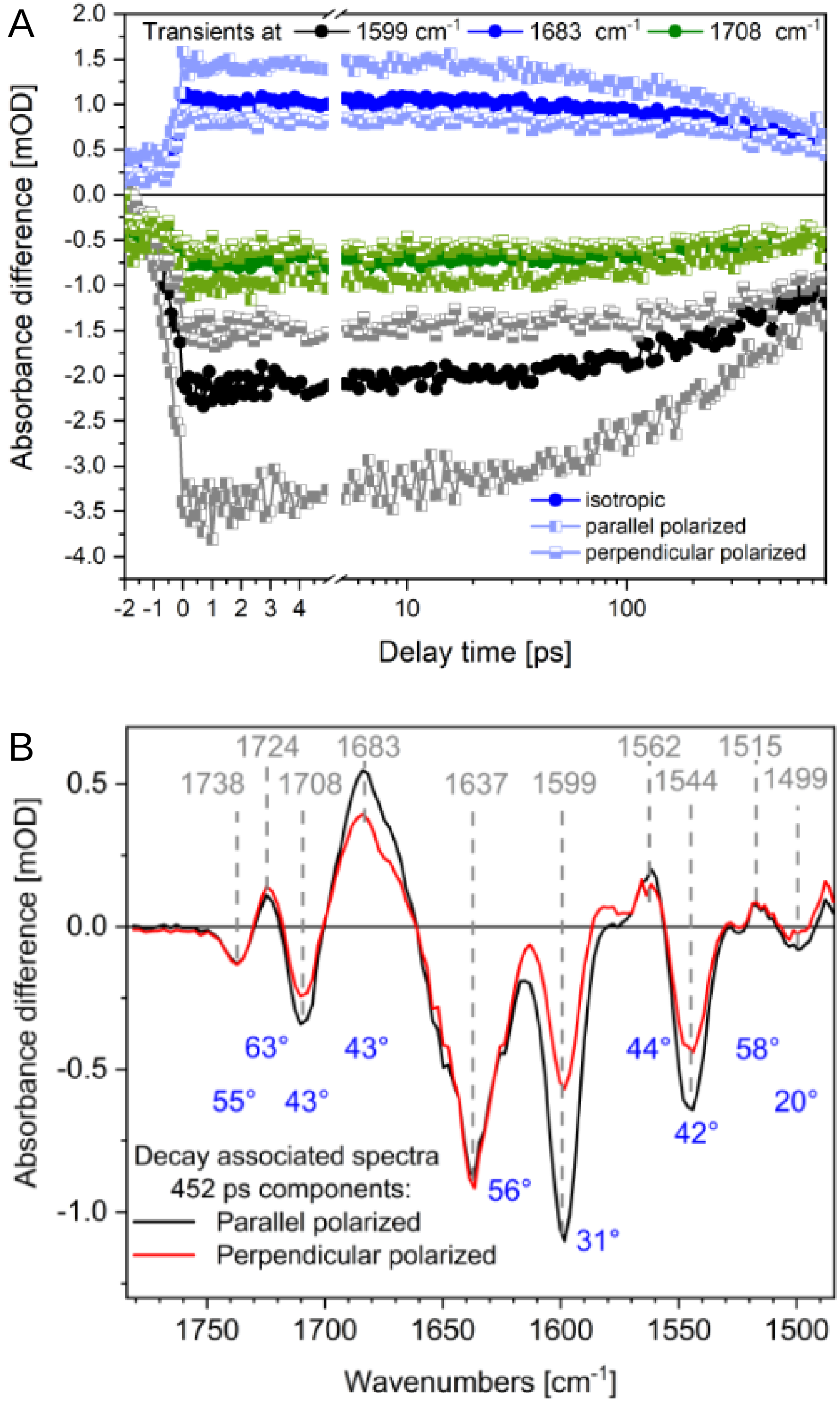

Figure S9. Ultrafast vibrational dynamics upon photoexcitation of the Y176H Pr state at 635 nm. (A) Decay associated spectra for isotropic polarization from a global fit with two exponential functions and a constant. (B) Selected transients at isotropic polarization, parallel polarization, and perpendicular polarization with respect to pump pulse polarization. All transients exhibit slow dynamics on a time-scale of hundreds of picoseconds.

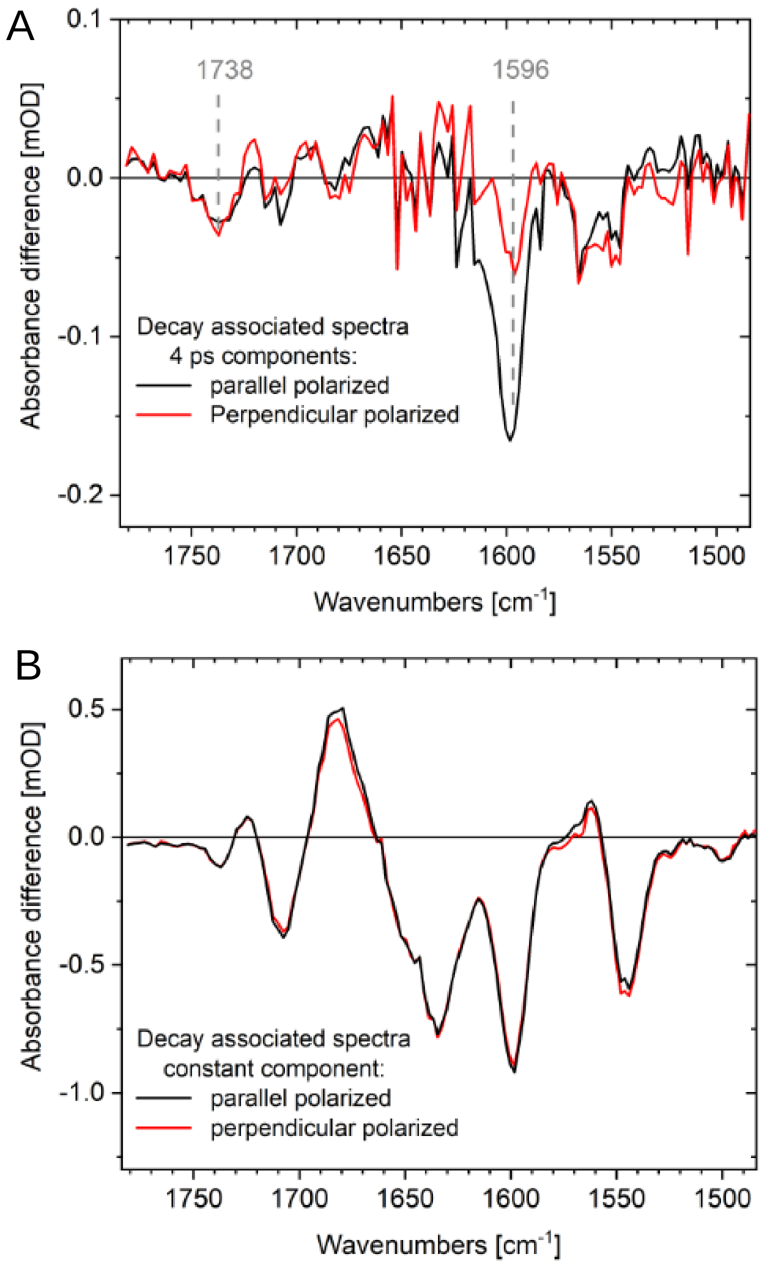

Figure S10. Polarization-resolved decay associated spectra from ultrafast vibrational dynamics upon photoexcitation of the Y176H Pr state at 635 nm. (A) Decay constant 4 ps; relevant spectral signatures occur at 1738 cm^{-1}, and 1596 cm^{-1}, with relative angles of about 54°, and about 0°, respectively. The negative signals indicate a change of ground state Pr absorption upon photoexcitation. (B) Constant component of the global fit representing dynamics longer than several hundreds of picoseconds. The absence of anisotropy, i.e. a difference in parallel and perpendicular polarized signals, show that rotational diffusion or other effects average out photoselection on this time-scale.

Table S1. List of primers used for mutagenesis in this study. Bold bases correspond to the alterations relative to the template sequences.

Template	Operation	Direction	Sequence 5'-3'
CPH1	Y176H	F	CGGGTGATGCTA**C**ACCGCTTTGATG
		R	GTCAAAGCCAGTCATACGGCGGACT
CPH1	N141R	F	**G**CGCTGCCCTGAATCGGTTG
		R	**G**GGCCATGTGATAAAAACCGAGG
CPH1	Removal of the 6His tag at the 3' end	F	TAAGCTTGGATCCCTGCAGGC
		R	CTCTTCTGCCTGGCGCAAAATGAG
CPH1	Addition of the 6His tag and TEV site at the 5' end	F	**GAAAATCTGTATTTTCAAGG C**CGCCACCACCGTACAACTCAGCG
		R	**GTGATGGTGATGGTGATGCAT** AGTTAATTTCTCCTCTTTAATGAATTCTGTGTG
Gm.PHYB	Y272H	F	**C**ATCGCTTTCACGAAGACGAGCACG
		R	CACCATCACGCGGTCATAGCCGG

Table S2. Data collection and refinement statistics (PDB code: 8RVX).

Data collection	
Space group	P 4_3 2_1 2
Cell dimensions	
a, b, c (Å)	135.87, 135.87, 355.64
α, β, γ (°)	90, 90, 90
Resolution (Å)	47.6-3.7 (3.8 - 3.7)[a]
R_{merge}	0.800 (3.702)
R_{pim}	0.157 (0.743)
$CC_{1/2}$	0.982 (0.398)
I/σI	6.3 (1.2)
Completeness (%)	99.3 (97.2)
Redundancy	25.8 (24.8)
Refinement	
Resolution (Å)	47.6-3.7 (3.8 - 3.7)[a]
No. of reflections	36277
R_{work}/R_{free} (%)	26.4 / 30.0
Overall B factor	115.8
Number of atoms	
Protein	15506
Ligand	172
Water	4
RMSD	
Bond length (Å)	0.009
Bond angles (°)	1.45
Ramachandran plot (%)[b]	
Favored	95.76
Allowed	4.24
Disallowed	0.00

a Highest resolution shell is shown in parentheses.

b Data are defined in the program Molprobity.[17]

Table S3. Overview of ^{13}C and ^{15}N chemical shifts of the u-[^{13}C,^{15}N]-PCB chromophore in Cph1-Y176H mutant. The ^{13}C and ^{15}N chemical shifts (in ppm) of the u-[^{13}C,^{15}N]-PCB-Cph1-WT sample in both Pr and Pfr states are listed for reference.

**PCB carbons**		**WT**		**Y176H**	**WT − Y176H Pr**	**Pfr − Pr WT**	**WT Pfr − Y176H Pr**
		**Pr**	**Pfr**	**Pr**			
**Ring *A***	C1	182.7/184.1	182.9	183.7	−0.3	−0.5	−0.8
	C2	37.1/38.0	37.3	37.1	−0.4	−0.3	+0.2
	$C2^1$	17.4	18.2	17.8	+0.4	+0.8	+0.4
	C3	53.0/53.6	54.3	54.1	−0.8	+1.0	+0.2
	$C3^1$	47.5	49.7	48.1	−0.6	+2.2	+1.6
	$C3^2$	20.5/23.5	21.2	21.0	+1.0	−0.8	+0.2
	C4	153.9	153.5	158.0	−4.1	−0.4	−4.5
A*–*B	C5	87.1	88.5	87.6	−0.5	+1.4	+0.9
**Ring *B***	C6	149.5	149.3	153.1	−3.6	-0.2	−3.8
	C7	125.5	126.3	127.9	−2.4	+0.8	−1.6
	$C7^1$	9.3	9.2	8.6	+0.7	−0.1	+0.6
	C8	144.8/145.8	143.8	146.8/148.4	−2.4	−1.4	−3.8
	$C8^1$	21.8/22.8	22.9	21.1	+1.2	+0.6	+1.8
	$C8^2$	42.9/41.4	41.8	40.8	+1.4	−0.4	+1.0
	$C8^3$	180.0/179.3	180.5	180.4/179.0	0.0	+0.8	+0.8
	C9	127.7	130.9	130.0	−2.3	+3.2	+0.9
B*–*C	C10	112.8	112.4	113.4	−0.6	−0.4	−1.0
**Ring *C***	C11	127.7	130.9	126.5	+1.2	+3.2	+4.4
	C12	145.2	145.8	145.9	−0.7	+0.6	−0.1
	$C12^1$	20.4	20.5	20.3	+0.1	+0.1	+0.2
	$C12^2$	38.1	38.4	38.7	−0.6	+0.3	−0.3
	$C12^3$	179.0	175.3	179.3	−0.3	−3.7	−4.0
	C13	126.4	130.7	122.6	+3.8	+4.3	+8.1
	$C13^1$	11.4	11.6	8.9	+2.5	+0.2	+2.7
	C14	145.9	152.0	142.3	+3.6	+6.1	+9.7
C*–*D	C15	93.2	91.6	94.3	−1.1	−1.6	−2.7
**Ring *D***	C16	145.9	151.6	142.3	+3.6	+5.7	+9.3
	C17	142.1	137.5	143.5	−1.4	−4.6	−6.0
	$C17^1$	9.1/10.7	10.0	8.2	+1.7	+0.1	+1.8
	C18	134.1	140.5	131.9	+2.2	+6.4	+8.6
	$C18^1$	16.5	15.7	16.7	−0.2	−0.8	−1.0
	$C18^2$	12.8/13.6	13.3	14.0	−0.8	+0.1	−0.7
	C19	172.7	169.1	172.1	+0.6	−3.6	−3.0
**PCB nitrogens**		**WT**		**Y176H**	**WT − Y176H Pr**	**Pfr − Pr WT**	**WT Pfr − Y176H Pr**
		**Pr**	**Pfr**	**Pr**			
	N21	159.4	158.2	161.5	−2.1	−1.2	−3.3
	N22	146.9	142.7	145.4	+1.4	−4.2	−2.7
	N23	157.0	155.6	155.3	+1.7	−1.4	+0.3
	N24	132.2	137.9	127.6	+4.6	+5.7	+10.3

Table S4. Selected structural parameters of the chromophore obtained by QMMM calculations.

	WT Pr-II	Y176HD	Y176HE	Y176HP
AB C-C bond / Å	1.435	1.450	1.451	1.445
AB C=C bond / Å	1.360	1.368	1.368	1.37
tilt A-B angle /degree	17.8	23.1	23.4	22.9
CD C-C bond / Å	1.440	1.435	1.434	1.439
CD C=C bond / Å	1.359	1.364	1.365	1.364
tilt C-D angle /degree	136.2	134.8	140.9	153.1
BC CCC angle /degree	132.2	132.3	132.6	133.0
Tilt angle C=O (D) /degree	87.1	79.9	85.5	95.5
Tilt angle C=O (A) /degree	106.2	94.0	90.8	94.5

Table S5. Experimental and calculated modes of WT Pr-II and Y176H in the region between 1650 and 1550 cm^{-1}.[a]

Pr-II WT			Y176H				
				**HSP**	**HSE**	**HSD**	
exp. ν (I_{ra})	calc. ν (I_{Ra})	mode	exp. ν (I_{Ra})	calc. ν (I_{Ra})	calc. ν (I_{Ra})	calc. ν (I_{Ra})	mode
1646 (w)	1638 (w)	A-B					
1631 (s)	1635 (s)	C-D	1650 (vs)	1629 (s)	1625 (s)	1622 (s)	C-D
	1630 (m)	D C=C	1640 (m)	1621 (m)	1622 (m)	1619 (m)	D C=C
			1606 (w)	1612 (w)	1615 (w)	1616 (w)	A-B, B-C
1608 (w)	1599 (w)	B-C	1587 (w)	1580 (w)	1583 (w)	1584 (w)	B-C, A-B
1570 (m)	1593 (m)	B,C NHip	1564 (m)	1555(m)	1557 (m)	1556 (m)	B, C NHip

[a] HSP, HSE, and HSD refer to the models of different protonation state of His176, i.e. double protonated (HSP) and single protonated at Nε (HSE) and Nδ (HSD); frequencies (ν) are given in cm^{-1}; intensities I_{Ra} (in parentheses) are expressed qualitatively as very strong (s), medium (m), and weak (w). Experimental and calculated data for Pr-II WT were taken from previous work.[15].

References

(1) Thakur, R. S., Kurur, N. D., and Madhu, P. K. (2006) Swept-frequency two-pulse phase modulation for heteronuclear dipolar decoupling in solid-state NMR. *Chem. Phys. Lett. 426*, 459–463.

(2) Takegoshi, K., Yano, T., Takeda, K., and Terao, T. (2001) Indirect high-resolution observation of 14N NMR in rotating solids [25]. *J. Am. Chem. Soc. 123*, 10786–10787.

(3) Van Rossum, B. J., Schulten, E. A. M., Raap, J., Oschkinat, H., and De Groot, H. J. M. (2002) A 3-D structural model of solid self-assembled chlorophyll a/H2O from multispin labeling and MAS NMR 2-D dipolar correlation spectroscopy in high magnetic field. *J. Magn. Reson. 155*, 1–14.

(4) Van Rossum, B. J., Förster, H., and De Groot, H. J. M. (1997) High-Field and High-Speed CP-MAS 13 C NMR Heteronuclear Dipolar-Correlation Spectroscopy of Solids with Frequency-Switched Lee-Goldburg Homonuclear Decoupling. *J. Magn. Reson. 124*, 516–519.

(5) Essen, L.-O., Mailliet, J., and Hughes, J. (2008) The structure of a complete phytochrome sensory module in the Pr ground state. *Proc. Natl. Acad. Sci. U. S. A. 105*, 14709–14714.

(6) Stöppler, D., Song, C., van Rossum, B. J., Geiger, M. A., Lang, C., Mroginski, M. A., Jagtap, A. P., Sigurdsson, S. T., Matysik, J., Hughes, J., and Oschkinat, H. (2016) Dynamic Nuclear Polarization Provides New Insights into Chromophore Structure in Phytochrome Photoreceptors. *Angew. Chemie - Int. Ed. 55*, 16017–16020.

(7) Matysik, J., Lang, C., Gartner, W., Rohmer, T., Essen, L.-O., and Hughes, J. (2008) Light-induced chromophore activity and signal transduction in phytochromes observed by 13C and 15N magic-angle spinning NMR. *Proc. Natl. Acad. Sci. 105*, 15229–15234.

(8) Fischer, A. J., Rockwell, N. C., Jang, A. Y., Ernst, L. A., Waggoner, A. S., Duan, Y., Lei, H., and Lagarias, J. C. (2005) Multiple roles of a conserved GAF domain tyrosine residue in cyanobacterial and plant phytochromes. *Biochemistry 44*, 15203–15215.

(9) Rohmer, T., Lang, C., Gärtner, W., Hughes, J., and Matysik, J. (2010) Role of the protein cavity in phytochrome chromoprotein assembly and double-bond isomerization: A comparison with model compounds. *Photochem. Photobiol. 86*, 856–861.

(10) Jähnigen, S., and Sebastiani, D. (2020) Carbon atoms speaking out: How the geometric sensitivity of13c chemical shifts leads to understanding the colour tuning of phycocyanobilin in cph1 and anpixj. *Molecules 25*, 1–21.

(11) Linke, M., Yang, Y., Zienicke, B., Hammam, M. A. S., Von Haimberger, T., Zacarias, A., Inomata, K., Lamparter, T., and Heyne, K. (2013) Electronic transitions and heterogeneity of the bacteriophytochrome Pr absorption band: An angle balanced polarization resolved femtosecond VIS pump-IR probe study. *Biophys. J. 105*, 1756–1766.

(12) Yang, Y., Linke, M., von Haimberger, T., Matute, R., González, L., Schmieder, P., and Heyne, K.

(2014) Active and silent chromophore isoforms for phytochrome Pr photoisomerization: An alternative evolutionary strategy to optimize photoreaction quantum yields. *Struct. Dyn. 1*, 014701.

(13) Rao, A. G., Wiebeler, C., Sen, S., Cerutti, D. S., and Schapiro, I. (2021) Histidine protonation controls structural heterogeneity in the cyanobacteriochrome AnPixJg2. *Phys. Chem. Chem. Phys. 23,* 7359–7367.

(14) Wiebeler, C., Rao, A. G., Gärtner, W., and Schapiro, I. (2019) The Effective Conjugation Length Is Responsible for the Red/Green Spectral Tuning in the Cyanobacteriochrome Slr1393g3. *Angew. Chemie - Int. Ed. 58*, 1934–1938.

(15) Velazquez Escobar, F., Lang, C., Takiden, A., Schneider, C., Balke, J., Hughes, J., Alexiev, U., Hildebrandt, P., and Mroginski, M. A. (2017) Protonation-dependent structural heterogeneity in the chromophore binding site of cyanobacterial phytochrome cph1. *J. Phys. Chem. B 121*, 47–57.

(16) Nagano, S., Guan, K., Shenkutie, S., Feiler, C., Weiss, M., Kraskov, A., Buhrke, D., Hildebrandt, P., and Hughes, J. (2020) Structural insights into photoactivation and signalling in plant phytochromes. *Nat. Plants 6,* 581–588.

(17) Williams, C. J., Headd, J. J., Moriarty, N. W., Prisant, M. G., Videau, L. L., Deis, L. N., Verma, V., Keedy, D. A., Hintze, B. J., Chen, V. B., Jain, S., Lewis, S. M., Arendall, W. B., Snoeyink, J., Adams, P. D., Lovell, S. C., Richardson, J. S., and Richardson, D. C. (2018) MolProbity: More and better reference data for improved all-atom structure validation. *Protein Sci. 27,* 293–315.
